# Integrated Analytical Assessment of Edible and Essential Oils: Thermal, Oxidative, Chromatographic and Spectroscopic Characterization

**DOI:** 10.3390/molecules31214026

**Published:** 2026-11-09

**Authors:** Francielly Anjolin Lescano, Flavio Santana Michels, Regiane Santana da Conceição Ferreira Cabanha, Elaine Silva de Padua Melo, Lincoln Carlos Silva de Oliveira, Rita de Cássia Avellaneda Guimarães, Ademir da Silva Alves Junior, Karine de Cássia Freitas, Valter Aragão do Nascimento

**Affiliations:** 1 Group of Spectroscopy and Bioinformatics Applied Biodiversity and Health (GEBABS), Graduate Program in Health and Development in the Central-West Region of Brazil, Federal University of Mato Grosso do Sul (UFMS), Campo Grande 79079-900, MS, Brazil; fran_anjolin@hotmail.com (F.A.L.); regianecabanha19@gmail.com (R.S.d.C.F.C.); elaine.melo@uems.br (E.S.d.P.M.); rita.guimaraes@ufms.br (R.d.C.A.G.); ademir.alves@ufms.br (A.d.S.A.J.); karine.freitas@ufms.br (K.d.C.F.); 2 Instituto de Física, Federal University of Mato Grosso do Sul (UFMS), Campo Grande 79070-900, MS, Brazil; flavio.michels@ufms.br; 3 Faculty of Medicine, State University of Mato Grosso do Sul, Av. Dom Antônio Barbosa, 4155—Vila Santo Amaro, Campo Grande 79115-898, MS, Brazil; 4 Chemistry Institute, Federal University of Mato Grosso do Sul (UFMS), Campo Grande 79070-900, MS, Brazil; lincoln.oliveira@ufms.br

**Keywords:** vegetable oils, essential oils, oxidative stability, differential scanning calorimetry

## Abstract

This study investigates the physicochemical, thermal, oxidative, and spectroscopic properties of vegetable and essential oils using an integrated multianalytical approach. Commercial samples of avocado oil, grape seed oil (samples A and B), sucupira oil (samples A and B), eucalyptus essential oil (samples A and B), and sweet orange essential oil (samples A and B) were evaluated by differential scanning calorimetry (DSC), gas chromatography (GC-FID), Rancimat oxidative stability analysis, UV–Vis absorption spectroscopy, and excitation–emission fluorescence spectroscopy. Samples A and B represent different commercial samples of the same oil type obtained from different commercial products. DSC analyses revealed distinct thermal transition profiles associated with lipid composition and molecular organization. Avocado oil exhibited the most complex crystallization behavior and the highest transition enthalpy, indicating greater thermal organization. Chromatographic analysis demonstrated predominance of monounsaturated fatty acids in avocado oil, especially oleic acid, whereas grape seed and sucupira oils showed elevated concentrations of polyunsaturated fatty acids, mainly linoleic acid. Oxidative stability analysis showed significant differences among the samples, with avocado oil presenting the highest induction period (12.38 h), while eucalyptus, orange, and sucupira oils exhibited very low oxidative stability. UV–Vis and fluorescence analyses demonstrated distinct spectral profiles related to pigment composition, antioxidant compounds, and oxidation state. Overall, the results confirmed strong agreement among thermal, chromatographic, oxidative, and spectroscopic analyses, demonstrating the applicability of integrated analytical approaches for comprehensive characterization of vegetable and essential oils.

## 1. Introduction

Vegetable oils are complex lipid matrices obtained from oilseed crops through mechanical pressing or solvent extraction processes. After extraction, refining procedures are often required to remove impurities such as free fatty acids, moisture, phospholipids, and suspended particles, which directly affect physicochemical quality, shelf life, and oxidative stability [1,2]. These oils are predominantly composed of triacylglycerols containing saturated fatty acids (SFAs), monounsaturated fatty acids (MUFAs), and polyunsaturated fatty acids (PUFAs), in addition to minor bioactive compounds such as tocopherols, tocotrienols, sterols, carotenoids, chlorophylls, and phenolic compounds, which contribute to antioxidant activity and nutritional value [1,2].

Essential oils, in contrast, are volatile mixtures biosynthesized by plants and chemically composed mainly of monoterpenes, sesquiterpenes, and oxygenated derivatives such as alcohols, aldehydes, ketones, esters, and lactones [3,4]. These compounds exhibit recognized antioxidant, antimicrobial, anti-inflammatory, and pharmacological activities, although their composition may vary substantially according to botanical species, geographical origin, climatic conditions, extraction methodology, and storage parameters [3,4].

Among natural oils of increasing industrial and pharmacological relevance, avocado, eucalyptus, sweet orange, sucupira, and grape seed oils stand out due to their distinct chemical compositions and functional properties. Avocado oil is characterized by elevated oleic acid concentrations together with antioxidant compounds such as tocopherols and carotenoids, which contribute to cardioprotective and anti-inflammatory effects [5,6,7]. Eucalyptus essential oil contains high levels of oxygenated monoterpenes, especially 1,8-cineole, being widely applied in pharmaceutical and respiratory formulations [8]. Sweet orange essential oil is rich in limonene and oxygenated derivatives associated with antioxidant and anxiolytic activities [9,10]. Sucupira oil (*Pterodon emarginatus* Vogel) has attracted growing attention because of the presence of diterpenes and sesquiterpenes associated with anti-inflammatory and antimicrobial activities [11,12]. Grape seed oil is recognized for its elevated linoleic acid and phenolic compound contents, presenting important nutritional and antioxidant potential, although its oxidative stability strongly depends on extraction conditions and degree of unsaturation [13,14].

The oxidative stability of edible oils is directly influenced by lipid composition, especially the degree of unsaturation and the presence of antioxidant constituents [2,15]. Polyunsaturated fatty acids are more susceptible to lipid peroxidation because bis-allylic hydrogen atoms facilitate free radical propagation reactions [15]. Consequently, understanding the relationship between fatty acid composition, thermal behavior, and oxidative resistance is essential for evaluating oil quality and industrial applicability.

Recent studies have demonstrated the importance of integrated analytical approaches for characterization of complex lipid systems. In this context, Melo et al. [16] established a relevant methodological framework by combining gas chromatography (GC), differential scanning calorimetry (DSC), oxidative stability analysis (Rancimat), UV–Vis spectroscopy, and fluorescence spectroscopy to evaluate the oxidative behavior of babassu oil. According to the authors, the integration of chromatographic, thermal, and spectroscopic techniques provides complementary information regarding molecular composition, thermal transitions, oxidative resistance, and degradation mechanisms.

Gas chromatography has been extensively employed for fatty acid identification and characterization of lipid matrices [17]. Differential scanning calorimetry enables investigation of crystallization and melting transitions, providing important information regarding lipid polymorphism, molecular organization, and thermal stability [18]. Oxidative stability analyses based on the Rancimat method are widely used for determining induction periods and resistance to accelerated oxidation conditions [19]. Complementarily, fluorescence spectroscopy has emerged as a highly sensitive analytical tool for detecting oxidative degradation products and natural fluorophores in edible oils [20]. UV–Vis absorption and other spectroscopic techniques have also been successfully applied to characterize oxidation processes, pigment composition, and related molecular changes in vegetable oils [21,22].

Several studies have individually applied these analytical techniques to different oil matrices. Salgado et al. [23] correlated oleic acid content with thermal stability in avocado oil using DSC and chromatographic analyses. Bail et al. [24] characterized grape seed oils through triacylglycerol composition, antioxidant capacity, and volatile compounds. Torres et al. [25] investigated the thermal and chromatographic characteristics of *Pterodon emarginatus* oil, identifying important bioactive constituents associated with oxidative behavior. Turek and Stintzing [26] demonstrated that essential oils rich in volatile compounds exhibit thermal and oxidative properties substantially different from those observed in edible vegetable oils. Similarly, Guerrero et al. [27] reported that eucalyptus essential oil presents narrow thermal transitions associated with its homogeneous terpene composition and elevated volatility.

The physicochemical and thermal properties of avocado oil are also strongly influenced by extraction methodology [28], while oxidative resistance under accelerated conditions is closely associated with fatty acid composition and activation energy [29]. In addition, the oxidative behavior of edible oils is highly dependent on the degree of unsaturation and antioxidant content [30]. Fluorescence spectroscopy has been successfully applied to correlate oxidative degradation and molecular changes in lipid systems [31,32], whereas studies involving vegetable oils rich in PUFA demonstrated that increasing unsaturation significantly reduces oxidative stability under thermal stress conditions [33]. Chromatographic methods have also proven effective for authenticity evaluation and quality control of avocado oils [34].

Despite the increasing number of studies involving vegetable and essential oils, most investigations remain limited to isolated analytical techniques or individual oil matrices. Comparative studies integrating chromatographic, thermal, oxidative, and spectroscopic analyses under standardized experimental conditions remain relatively limited, particularly for the simultaneous evaluation of fixed vegetable oils and volatile essential oils with markedly different chemical compositions.

The selection of oil matrices evaluated in this study was strategically based on their distinct chemical compositions, physical states, and industrial relevance. Avocado (*Persea gratissima*) and grape seed (*Vitis vinifera*) oils were selected as representative fixed vegetable oils with contrasting fatty acid profiles—avocado oil being predominantly monounsaturated (rich in oleic acid) and grape seed oil being highly polyunsaturated (rich in linoleic acid). Eucalyptus (*Eucalyptus globulus*) and sweet orange (*Citrus aurantium dulcis*) essential oils were included as representative volatile, non-lipid terpene-rich matrices to provide a direct analytical contrast to fixed vegetable oils. Finally, sucupira (*Pterodon emarginatus*) oil was chosen as a unique native Brazilian plant matrix containing a complex mixture of diterpenic compounds, phenolic constituents, and unsaturated fatty acids. Including such chemically diverse fixed, volatile, and bioactive-rich oils allowed for a comprehensive evaluation of the diagnostic capacity of the integrated analytical approach across distinct lipid and volatile systems.

Based on this gap, we hypothesized that the distinct chemical compositions and molecular characteristics of vegetable and essential oils would be reflected in their fatty acid profiles, thermal transitions, oxidative stability, and spectroscopic characteristics, and that the complementary use of these analytical techniques would provide a more comprehensive basis for characterizing and comparing chemically distinct oil matrices.

Therefore, the primary objective of this study was to characterize and comparatively evaluate avocado (*Persea gratissima*), eucalyptus (Eucalyptus globulus), sweet orange (*Citrus aurantium dulcis*), sucupira (*Pterodon emarginatus*), and grape seed (*Vitis vinifera*) oils using an integrated analytical approach. Specifically, the study aimed to (i) characterize the fatty acid composition by gas chromatography (GC-FID); (ii) investigate thermal behavior through differential scanning calorimetry (DSC); (iii) evaluate oxidative stability using the Rancimat method; (iv) characterize the UV–Vis absorption and excitation–emission fluorescence profiles of the oils; and (v) compare the analytical profiles obtained for the different oil matrices under standardized experimental conditions. The integrated interpretation of these complementary analytical data was intended to provide a comprehensive physicochemical characterization and comparison of the selected vegetable and essential oils.

## 2. Results

Results follow the sequence of the preceding methodological sections. These methods allow for the assessment of fatty acid profiles and oxidative stability across all oil samples.

### 2.1. Thermal Behavior and Phase Transitions by Differential Scanning Calorimetry

**Figure 1:**
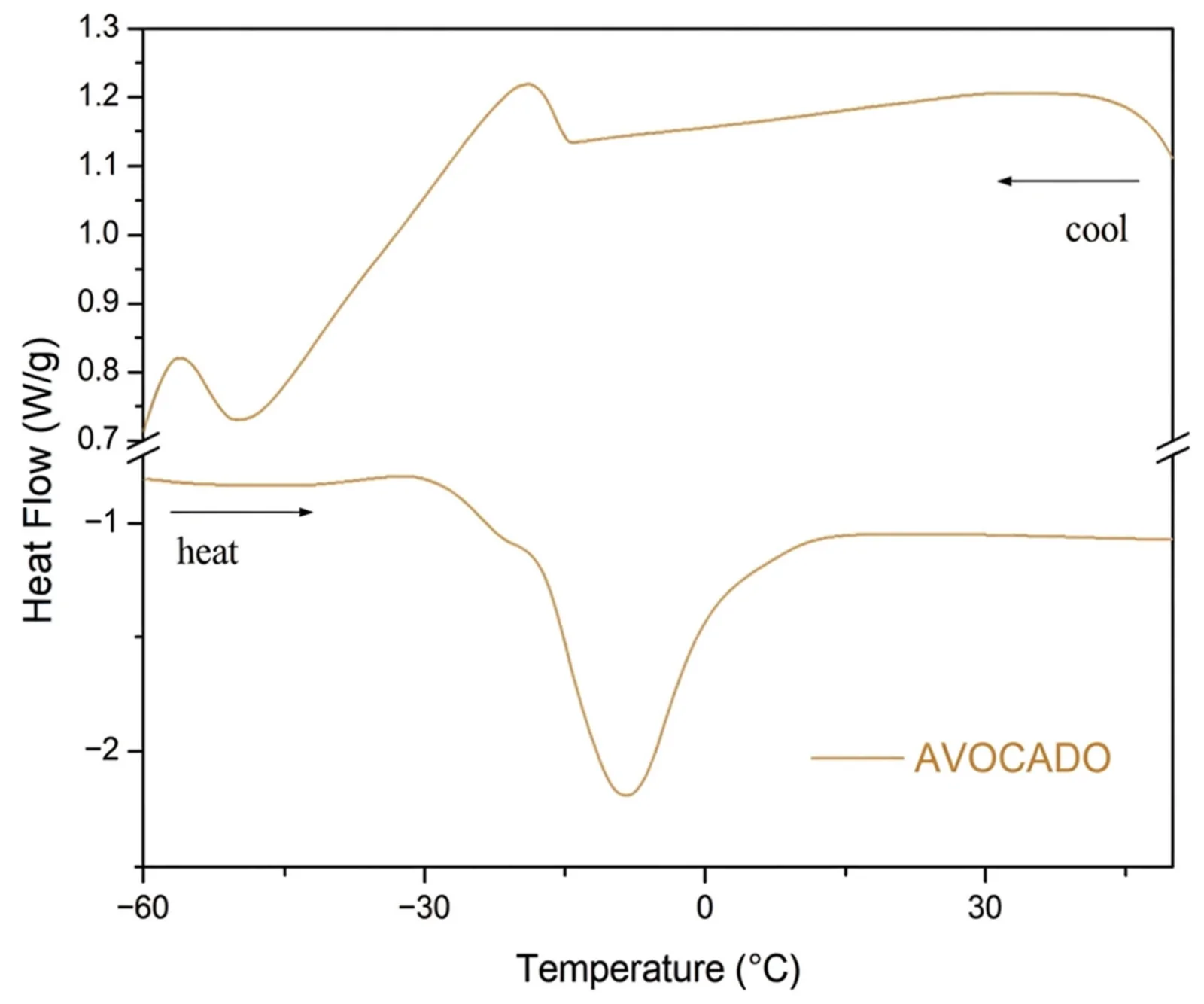
Differential Scanning Calorimetry (DSC) curves show the crystallization (upper curve) and melting behavior (lower curve) of avocado oil.

**Figure 2:**
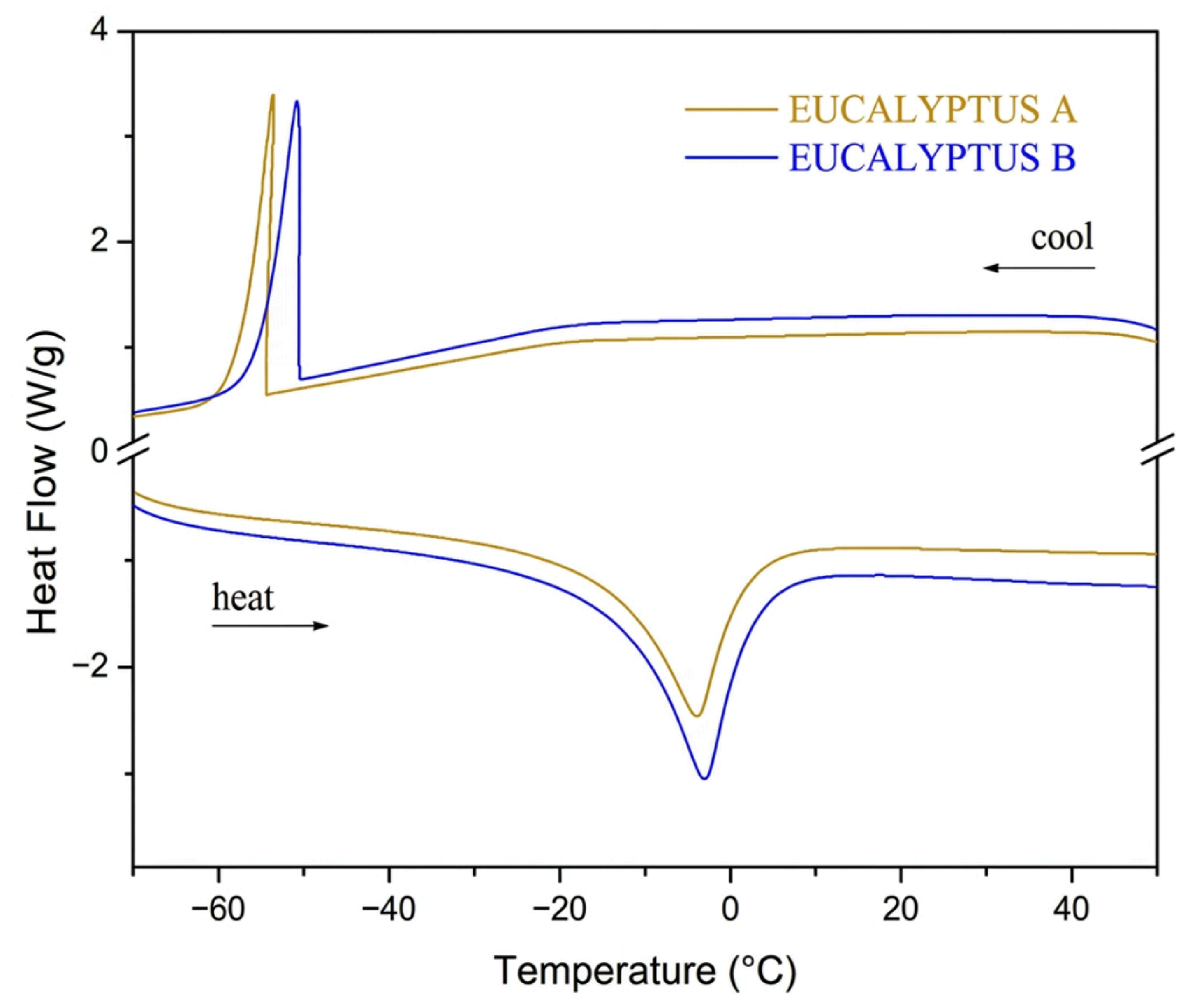
Differential Scanning Calorimetry (DSC) curves show the crystallization (upper curve) and melting behavior (lower curve) of eucalyptus oil samples: (orange) EUCALYPTUS A; (blue) EUCALYPTUS B.

**Figure 3:**
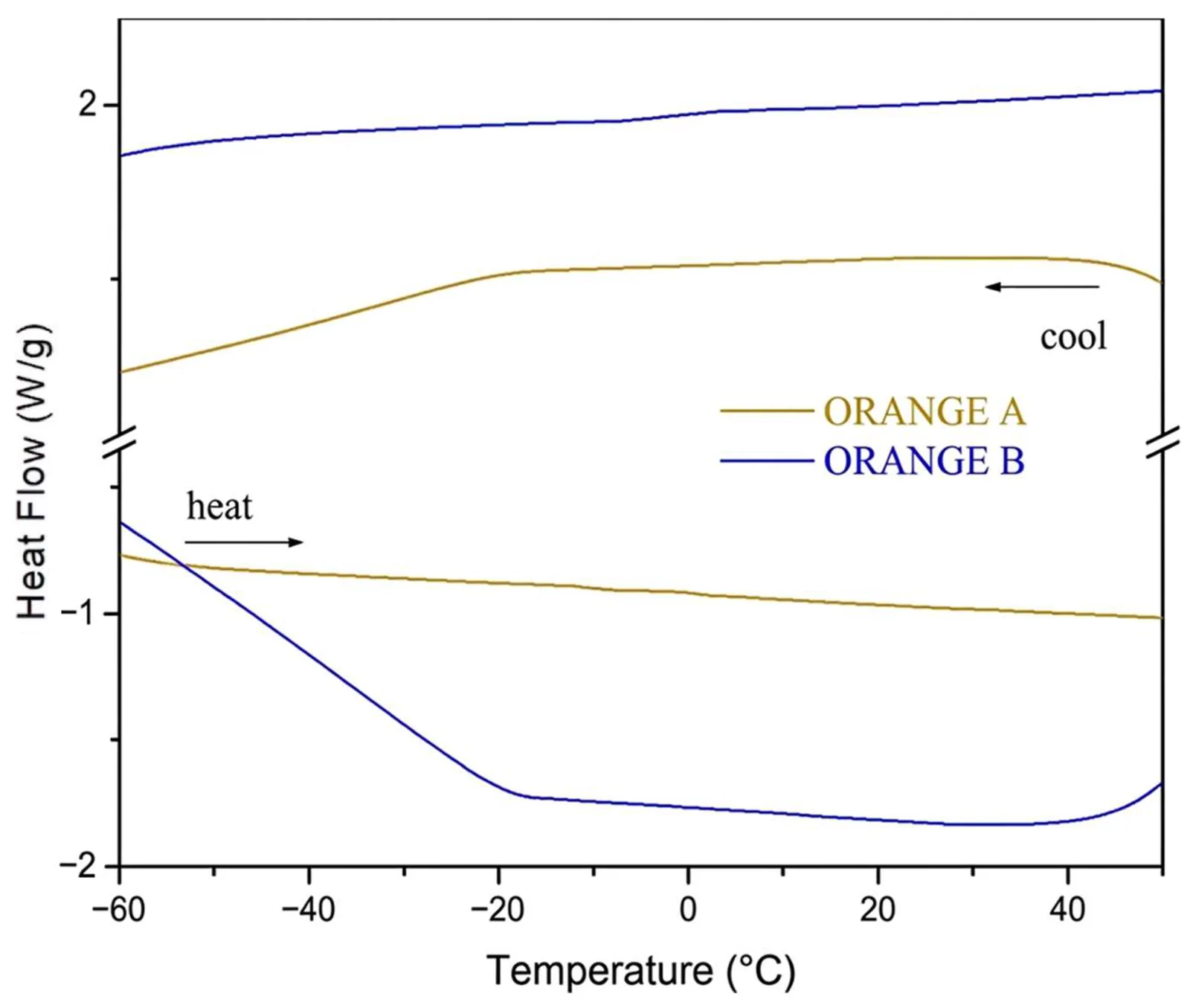
Differential Scanning Calorimetry (DSC) curves show the crystallization (upper curve) and melting behavior (lower curve) of orange oil samples: (orange) ORANGE A; (blue) ORANGE B.

**Figure 4:**
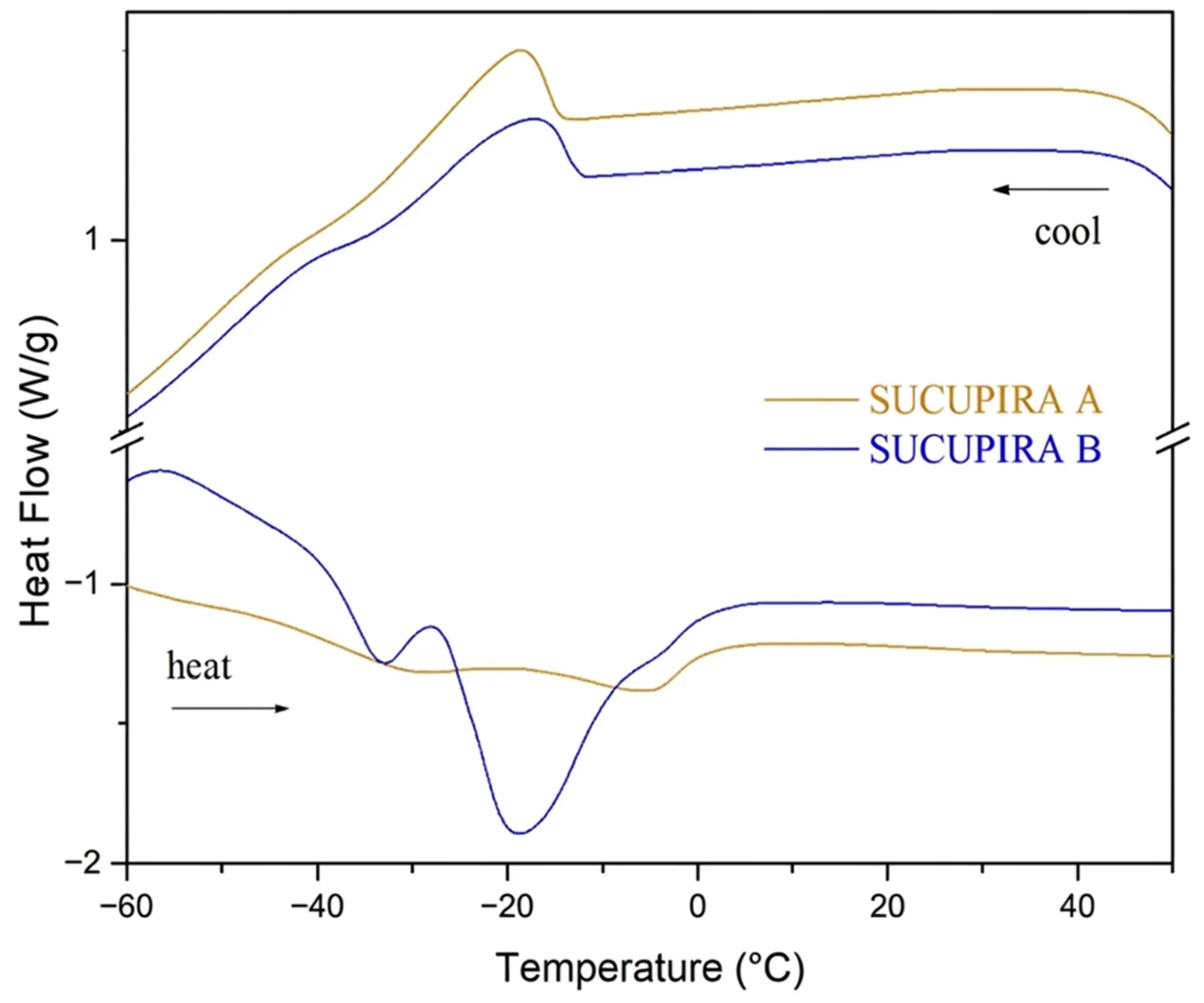
Differential Scanning Calorimetry (DSC) curves show the crystallization (upper curve) and melting behavior (lower curve) of sucupira oil samples: (orange) SUCUPIRA A; (blue) SUCUPIRA B.

**Figure 5:**
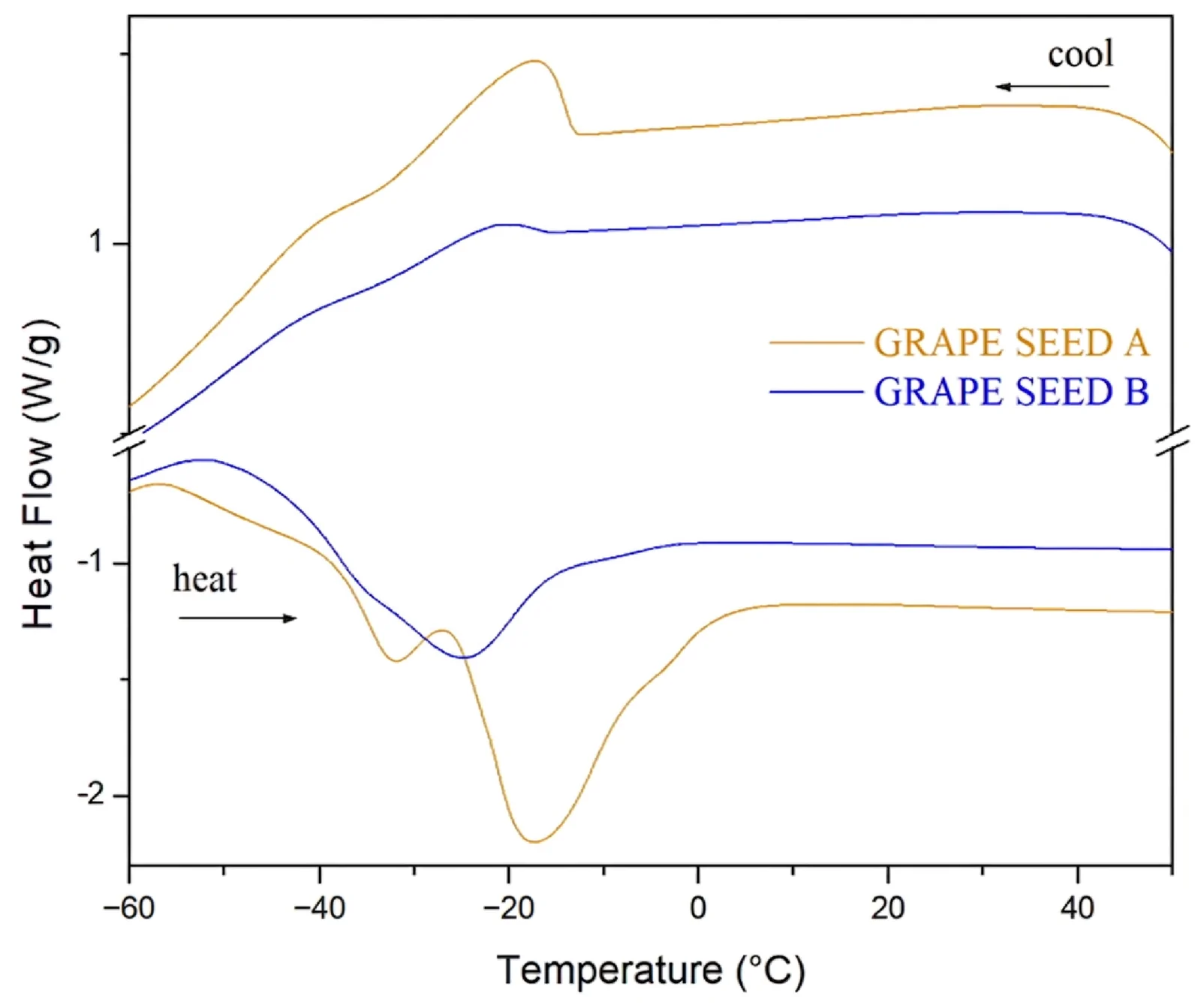
Differential Scanning Calorimetry (DSC) curves show the crystallization (upper curve) and melting behavior (lower curve) of grape seed oil samples: (orange) GRAPE SEED A; (blue) GRAPE SEED B.

The DSC curves obtained for the evaluated vegetable and essential oils are presented in Figure 1, Figure 2, Figure 3, Figure 4 and Figure 5, while the corresponding thermal transition parameters, including initial temperature (Tinitial), peak temperature (Tpeak), and enthalpy values (ΔH) for both cooling and heating cycles, are summarized in Table 1.

As shown in Figure 1, avocado oil exhibited two distinct exothermic transitions during the cooling cycle, indicating a multi-step crystallization process associated with the presence of different triacylglycerol fractions. In contrast, eucalyptus essential oils A and B (Figure 2), sweet orange essential oils A and B (Figure 3), sucupira oils A and B (Figure 4), and grape seed oils A and B (Figure 5) predominantly displayed a single exothermic transition under the same experimental conditions, suggesting a more homogeneous crystallization behavior.

Despite belonging to the same botanical species, the commercial samples exhibited noticeable differences in peak intensity, initial temperature, and enthalpy values, indicating variations in chemical composition and thermal behavior potentially associated with extraction methods, raw material origin, processing conditions, storage, and oxidative stability. The subsequent heating curves revealed characteristic endothermic melting events specific to each oil type and commercial sample, reflecting differences in fatty acid composition, volatile constituents, and triacylglycerol distribution (Table 1).

### 2.2. Fatty Acid Profiling by Gas Chromatography

It should be emphasized that eucalyptus oils A and B, as well as orange oils A and B, exhibited very similar fatty acid compositions and percentage distributions. Therefore, due to the minimal compositional differences observed between samples from the same botanical source, only a single representative value was considered for these oils in Table 2.

Table 2 presents the fatty acid (FA) composition of the analyzed oils obtained by Gas Chromatography (GC). Among the saturated fatty acids (SFA), grape seed oil B exhibited the lowest concentration (11.17%), followed by avocado oil (12.52%), grape seed oil A (14.83%), sucupira oil B (15.02%), and sucupira oil A (21.97%). In contrast, the essential oils showed extremely high proportions of saturated compounds, with orange oils A and B presenting 99.02% and eucalyptus oils A and B exhibiting 99.97%.

For monounsaturated fatty acids (MUFA), eucalyptus oils A and B showed the lowest concentration (0.03%), followed by orange oils A and B (0.81%). Higher MUFA concentrations were observed for grape seed oil B (14.11%), sucupira oil B (25.61%), sucupira oil A (26.24%), and grape seed oil A (28.36%), while avocado oil exhibited the highest MUFA content among all analyzed samples (63.15%), mainly associated with oleic acid.

Regarding polyunsaturated fatty acids (PUFA), orange oils A and B presented the lowest concentration (0.19%), followed by avocado oil (20.16%). Elevated PUFA concentrations were observed for sucupira oil A (51.24%), grape seed oil A (56.63%), sucupira oil B (59.37%), and grape seed oil B (69.85%), the latter presenting the highest degree of polyunsaturation among all analyzed samples.

According to Table 2, sucupira essential oils A and B, as well as grape seed vegetable oils A and B, exhibited higher concentrations of linoleic acid (C18:2n6c), followed by elaidic acid (C18:1n9t), palmitic acid (C16:0), and α-linolenic acid (C18:3n3). In addition, Avocado oil exhibited predominance of oleic acid (C18:1n9c), followed by linoleic acid (C18:2n6c) and palmitic acid (C16:0).

In contrast, eucalyptus essential oil showed elevated concentrations of the saturated fatty acids caproic acid (C6:0) and butyric acid (C4:0). Similarly, the fatty acid composition of sweet orange essential oil was comparable to that observed for eucalyptus oil, additionally presenting lauric acid (C12:0) among its constituents.

It should be noted that the summed percentages of SFA, MUFA, and PUFA do not necessarily reach 100% for all samples because the GC-FID analysis represents only the fatty acids detected, identified, and quantified under the chromatographic conditions employed. For avocado oil and grape seed oil B, the respective sums were 95.83% and 95.13%, as calculated from the values reported in Table 2. The remaining fractions were not attributed to specific compounds because they were not individually identified or quantified in the present analysis. Thus, these differences should not be interpreted as analytical errors, but rather as fractions outside the specific fatty-acid components identified and quantified within the scope of the chromatographic method.

### 2.3. Oxidative Stability and Induction Period Evaluation

**Figure 6:**
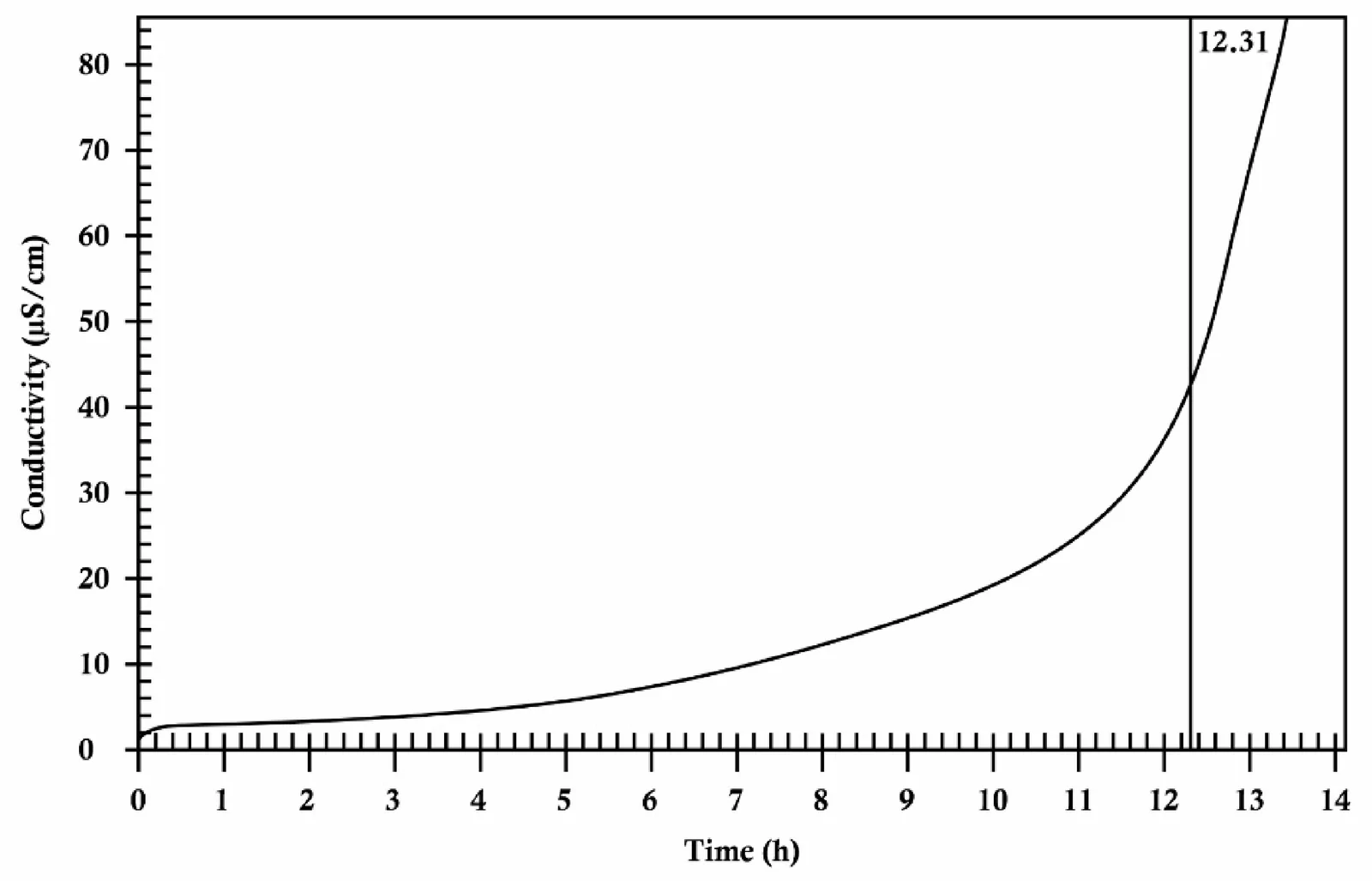
Conductivity (µS/cm) versus time (hours) determined by the Rancimat method. Oxidation stability of avocado oil at 110 °C. The induction time of avocado oil was 12.31 h.

**Figure 7:**
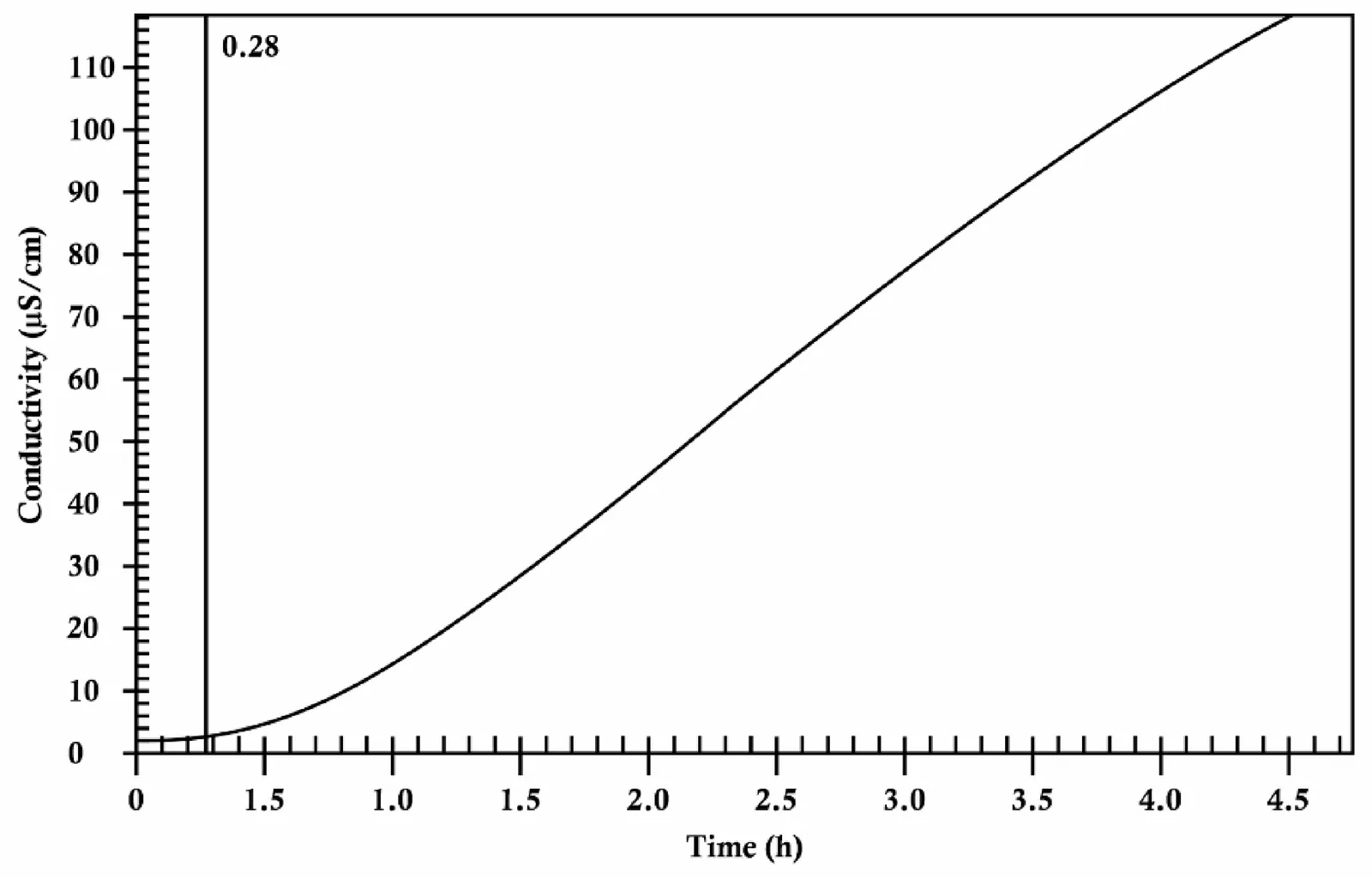
Conductivity (µS/cm) versus time (hours) determined by the Rancimat method. Oxidation stability of eucalyptus oil A and B at 110 °C. The induction time of both eucalyptus oils were 0.28 h.

**Figure 8:**
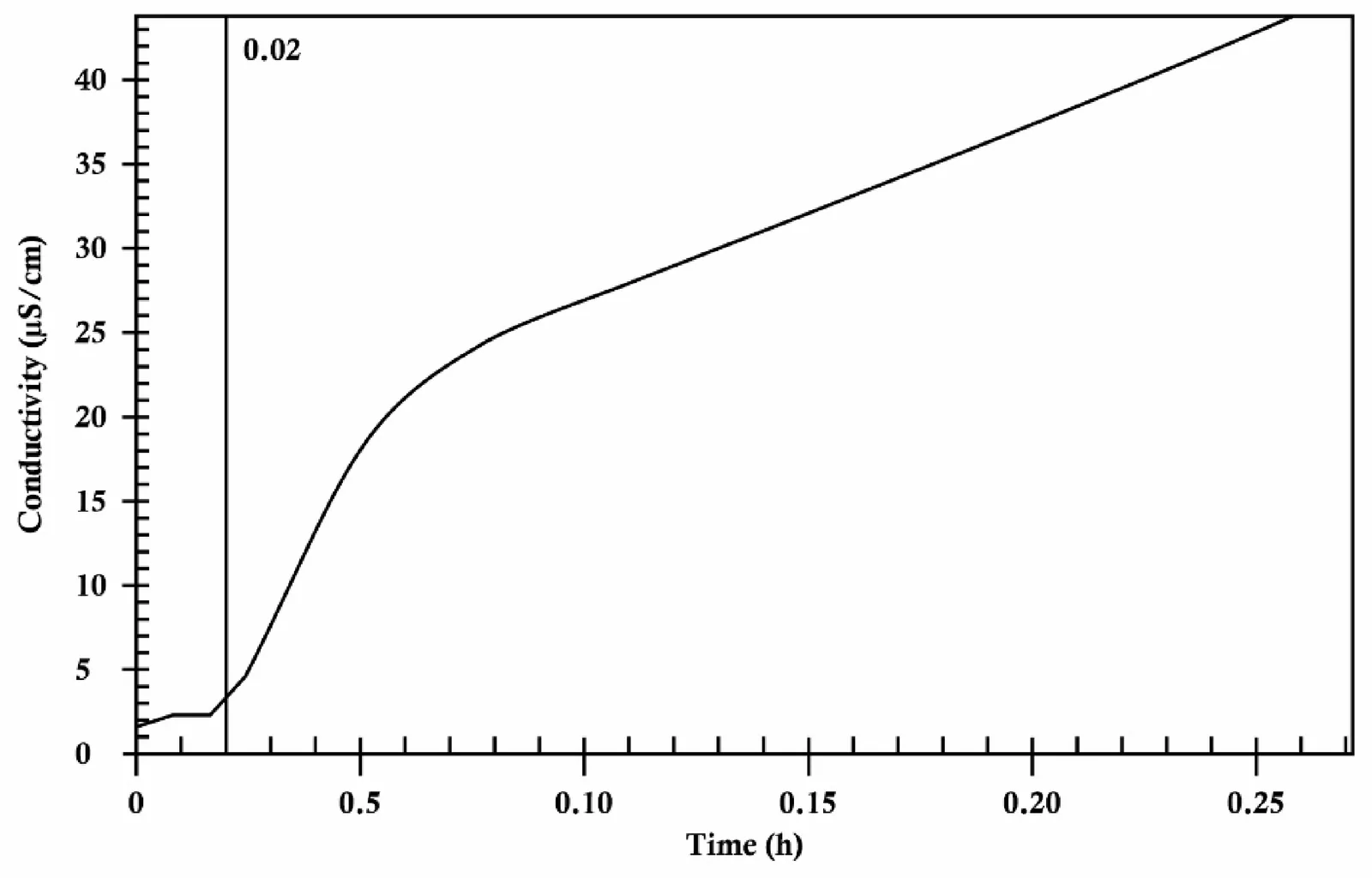
Conductivity (µS/cm) versus time (hours) determined by the Rancimat method. Oxidation stability of orange oil at 110 °C. The induction time of both orange oil was 0.02 h.

**Figure 9:**
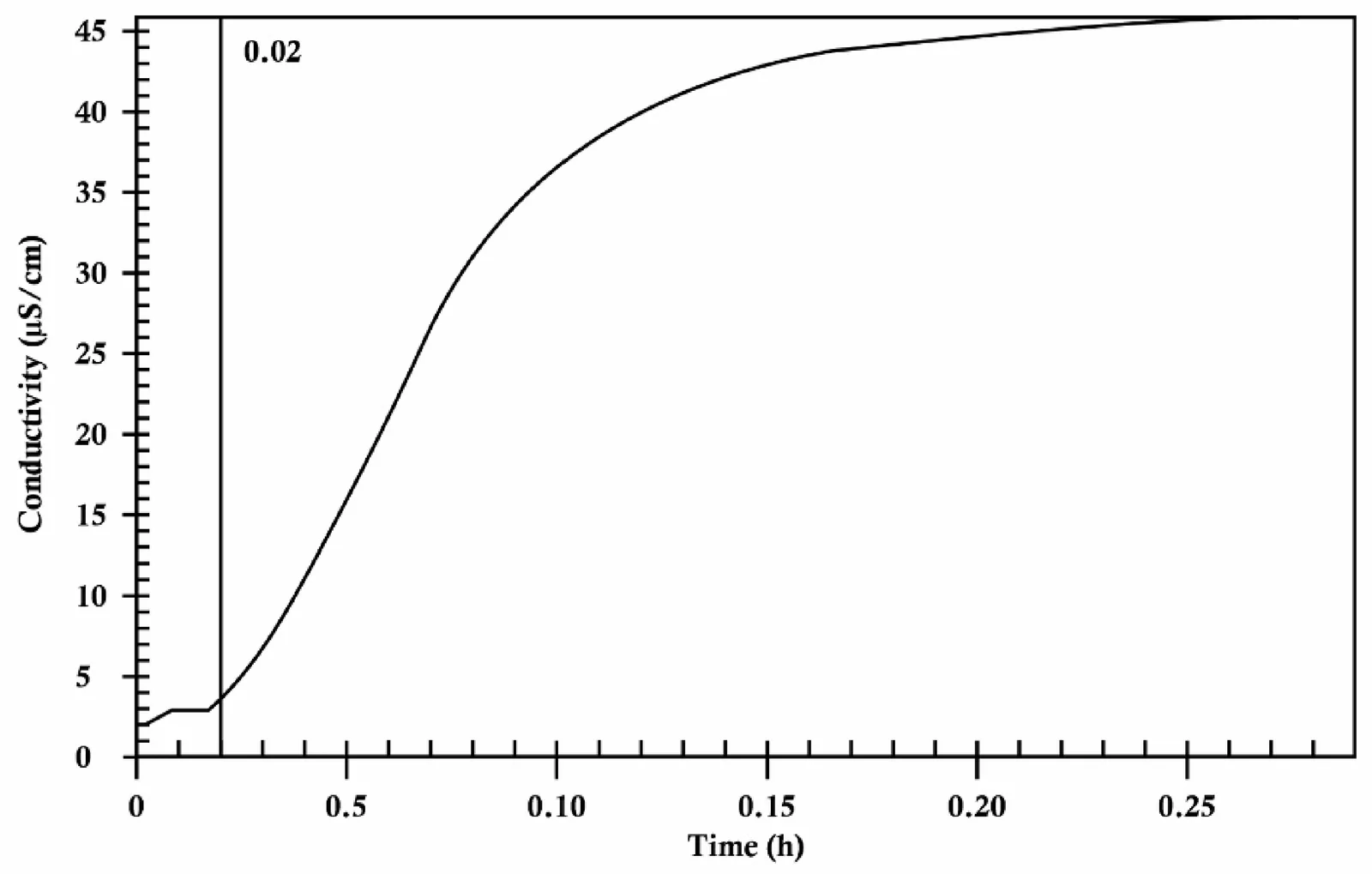
Conductivity (µS/cm) versus time (hours) determined by the Rancimat method. Oxidation stability of Sucupira A oil at 110 °C. The induction time of sucupira A oil was 0.02 h.

**Figure 10:**
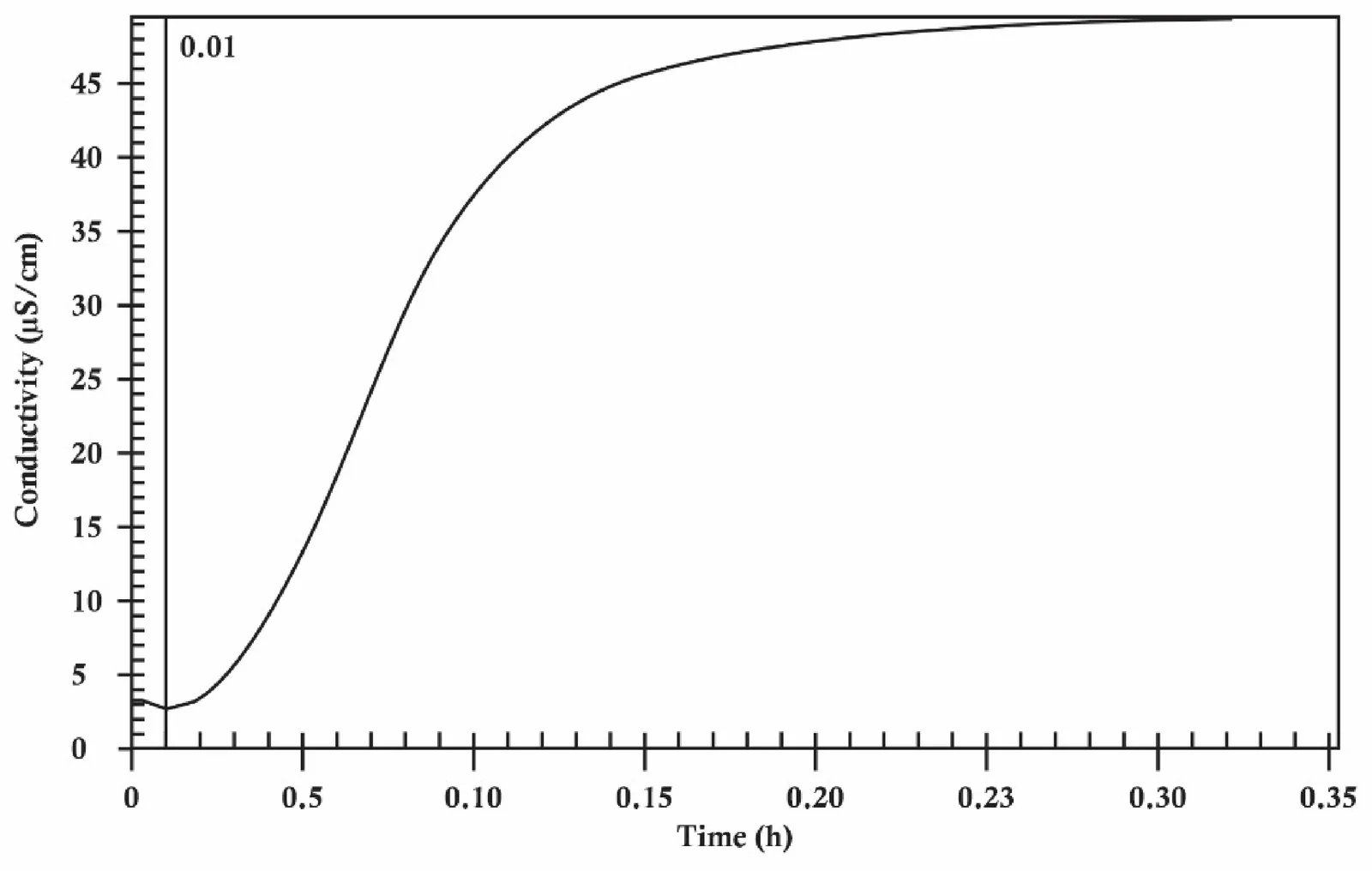
Conductivity (µS/cm) versus time (hours) determined by the Rancimat method. Oxidation stability of Sucupira B oil at 110 °C. The induction time of Sucupira B was 0.01 h.

**Figure 11:**
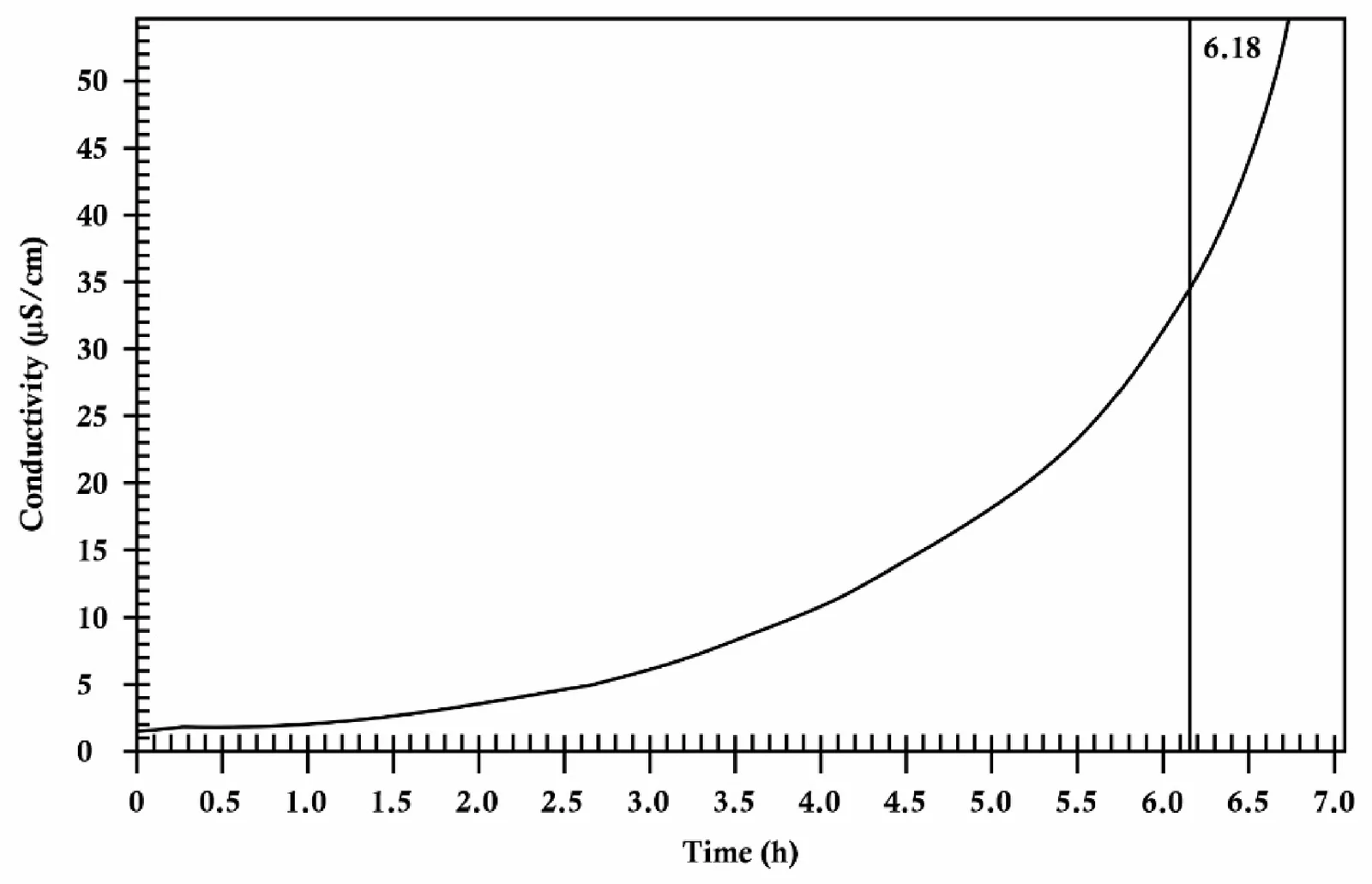
Conductivity (µS/cm) versus time (hours) determined by the Rancimat method. Oxidation stability of Grape seed oil A oil at 110 °C. The induction time of Grape seed oil A was 0.02 h.

**Figure 12:**
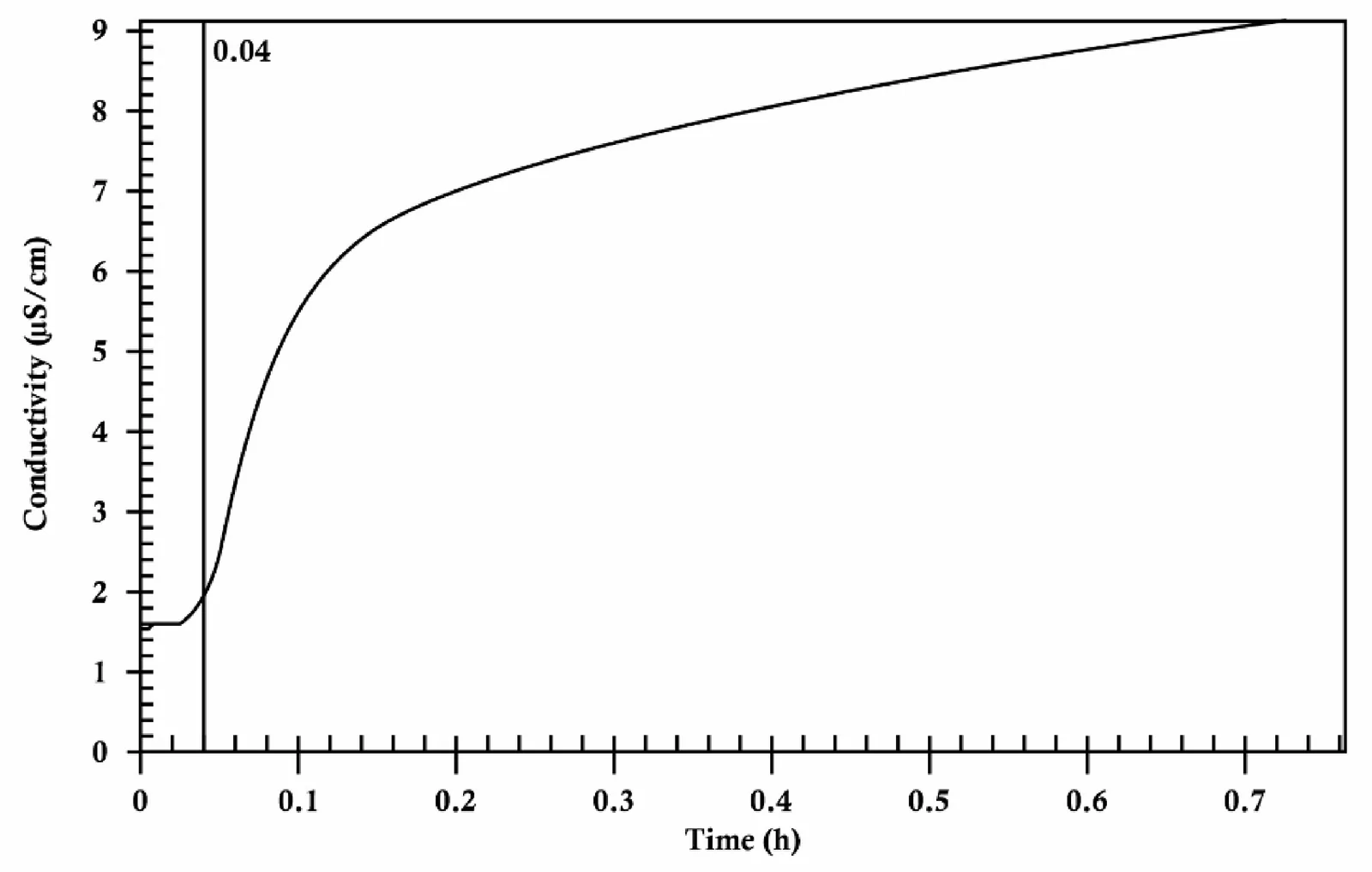
Conductivity (µS/cm) versus time (hours) determined by the Rancimat method. Oxidation stability of Grape seed oil B oil at 110 °C. The induction time of Grape seed oil B was 0.04 h.

The oxidative stability index (OSI), expressed as induction period time, for the evaluated oils is presented in Table 3. Additionally, the conductivity profiles as a function of induction time for the analyzed samples are shown in Figure 6, Figure 7, Figure 8, Figure 9, Figure 10, Figure 11 and Figure 12. For samples exhibiting identical induction times and overlapping conductivity profiles, only one representative graph was included.

According to the obtained results, the mean induction period was 12.38 h for avocado oil, 0.28 h for eucalyptus essential oils A and B, 0.02 h for sweet orange essential oils A and B, 0.02 h for sucupira oil A, 0.01 h for sucupira oil B, 6.18 h for grape seed oil A, and 0.04 h for grape seed oil B.

Among the analyzed samples, sucupira oil B (Figure 10) exhibited the shortest induction period, indicating the lowest oxidative stability, followed by sweet orange essential oils and sucupira oil A (0.02 h) (Figure 9), and grape seed oil B (0.04 h) (Figure 12). In contrast, avocado oil showed the highest oxidative stability, with an induction period of 12.38 h (Figure 6), followed by grape seed oil A (6.18 h, Figure 11) and eucalyptus essential oils A and B (0.28 h, Figure 7).

The markedly higher oxidative stability observed for avocado oil may be associated with its elevated content of monounsaturated fatty acids and natural antioxidant compounds, whereas the lower stability of the essential oils is likely related to their high concentration of volatile terpenoid constituents susceptible to thermal oxidation. Furthermore, although eucalyptus oils A and B, as well as orange oils A and B, originated from different commercial suppliers, the individual analyses of each pair yielded exactly the same induction-period value under the experimental conditions employed. Therefore, only one representative conductivity curve was presented for each pair of samples.

### 2.4. UV–Vis Molecular Absorption Profiles

**Figure 13:**
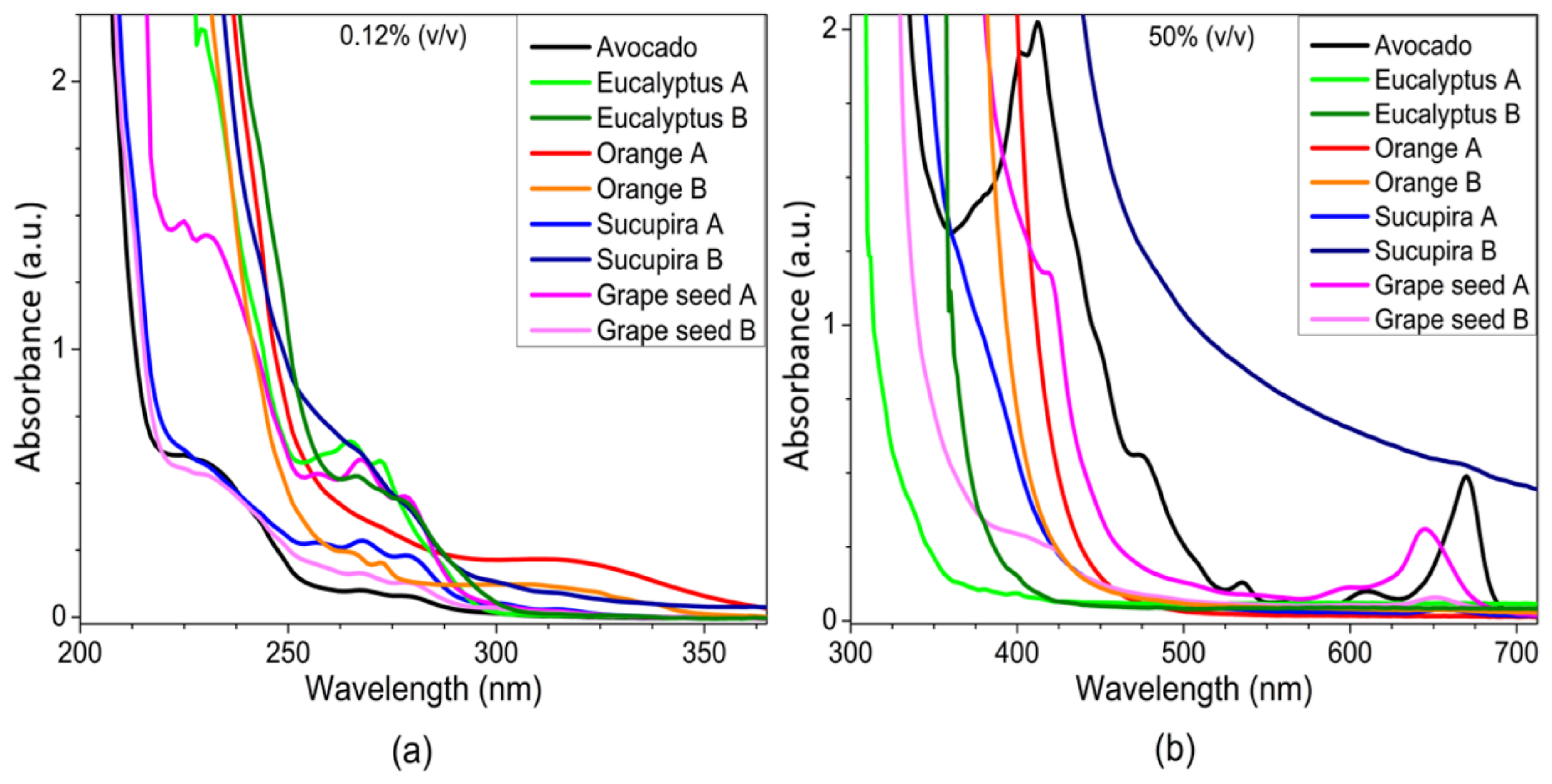
UV–Vis absorption spectra of avocado and grape seed vegetable oils and eucalyptus, orange, and sucupira essential oils recorded in the wavelength range of 250–750 nm. For the optical measurements, the samples were diluted in HPLC-grade hexane (99.9%, Sigma-Aldrich, St. Louis, MO, USA) at concentrations of (**a**) 0.12% (*v*/*v*) and (**b**) 50% (*v*/*v*).

Figure 13 presents the UV–Vis absorption spectra of the evaluated oil samples, including the commercial samples identified as A and B for eucalyptus, orange, sucupira, and grape seed oils. For the essential oils, eucalyptus A and eucalyptus B exhibited similar spectral contours, with characteristic absorption bands predominantly between approximately 225 and 280 nm. Orange essential oils A and B also displayed absorption bands within this same spectral region, indicating comparable compositions associated with terpenoid and unsaturated constituents typical of citrus essential oils. Sucupira essential oil A exhibited absorption bands in the same ultraviolet region, whereas sucupira essential oil B did not present well-defined absorption bands under the evaluated experimental conditions, suggesting compositional differences between the two commercial samples.

Regarding the vegetable oils, distinct spectral behaviors were observed between the analyzed commercial brands. Avocado oil exhibited the most intense absorption bands, predominantly distributed between 410 and 670 nm. Grape seed oil A showed intense absorption bands between 420 and 648 nm, together with less intense bands in the region from 225 to 265 nm, with the visible-region bands being predominant. In contrast, grape seed oil B did not exhibit significant absorption bands throughout the analyzed spectral range.

Therefore, the results demonstrate noticeable spectral differences between samples A and B of the same botanical origin, particularly for sucupira and grape seed oils, reinforcing the influence of commercial source, processing conditions, and possible compositional variability on the optical properties of the evaluated oils.

### 2.5. Excitation–Emission Fluorescence Characterization

**Figure 14:**
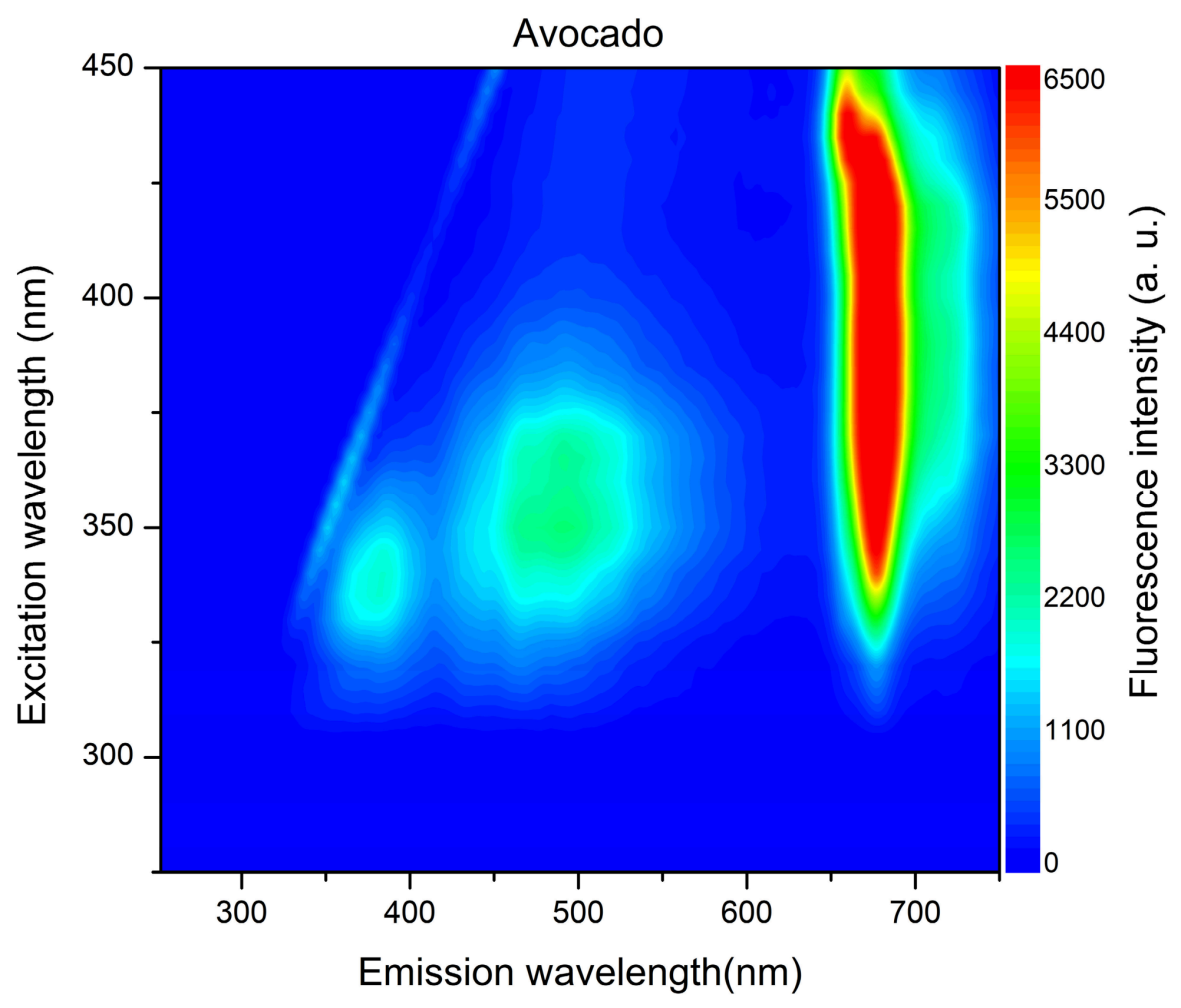
Excitation–emission maps of avocado oil obtained in the excitation wavelength range of 250–550 nm and emission range of 250–750 nm.

Figure 14 presents the excitation–emission map of avocado vegetable oil recorded in the excitation wavelength range of 250–550 nm and emission range of 250–750 nm. Four main fluorescence regions were identified. The first emission region was centered around 340 nm, associated with excitation wavelengths between approximately 325 and 450 nm. A second fluorescence band was observed for excitation wavelengths from 325 to 350 nm and emission between 355 and 400 nm. Another distinct region appeared with excitation ranging from approximately 330 to 375 nm and emission extending from 450 to 530 nm. Finally, a broader fluorescence band was detected at excitation wavelengths between 320 and 450 nm, accompanied by emission in the 650–730 nm region.

**Figure 15:**
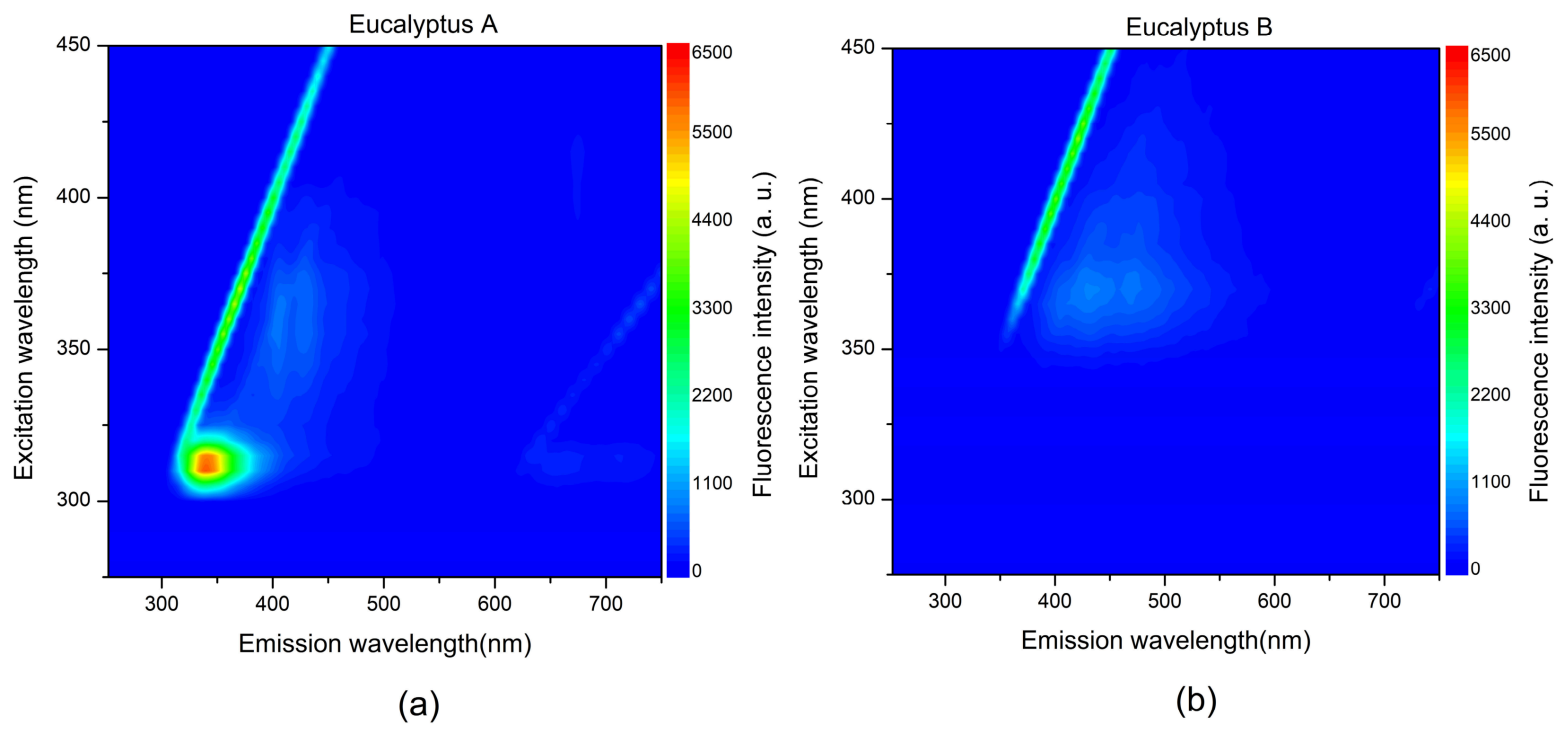
Excitation–emission maps of eucalyptus essential oils obtained in the excitation wavelength range of 250–550 nm and emission range of 250–750 nm: (**a**) EUCALYPTUS A; (**b**) EUCALYPTUS B.

Figure 15 shows the excitation–emission maps of eucalyptus essential oils A and B obtained in the excitation range of 250–550 nm and emission range of 250–750 nm. Both samples displayed similar fluorescence behavior, characterized by excitation bands mainly distributed between 300 and 450 nm, with emission signals extending approximately from 320 to 550 nm. Eucalyptus A exhibited a more intense fluorescence region at lower emission wavelengths, whereas Eucalyptus B showed a broader emission profile.

**Figure 16:**
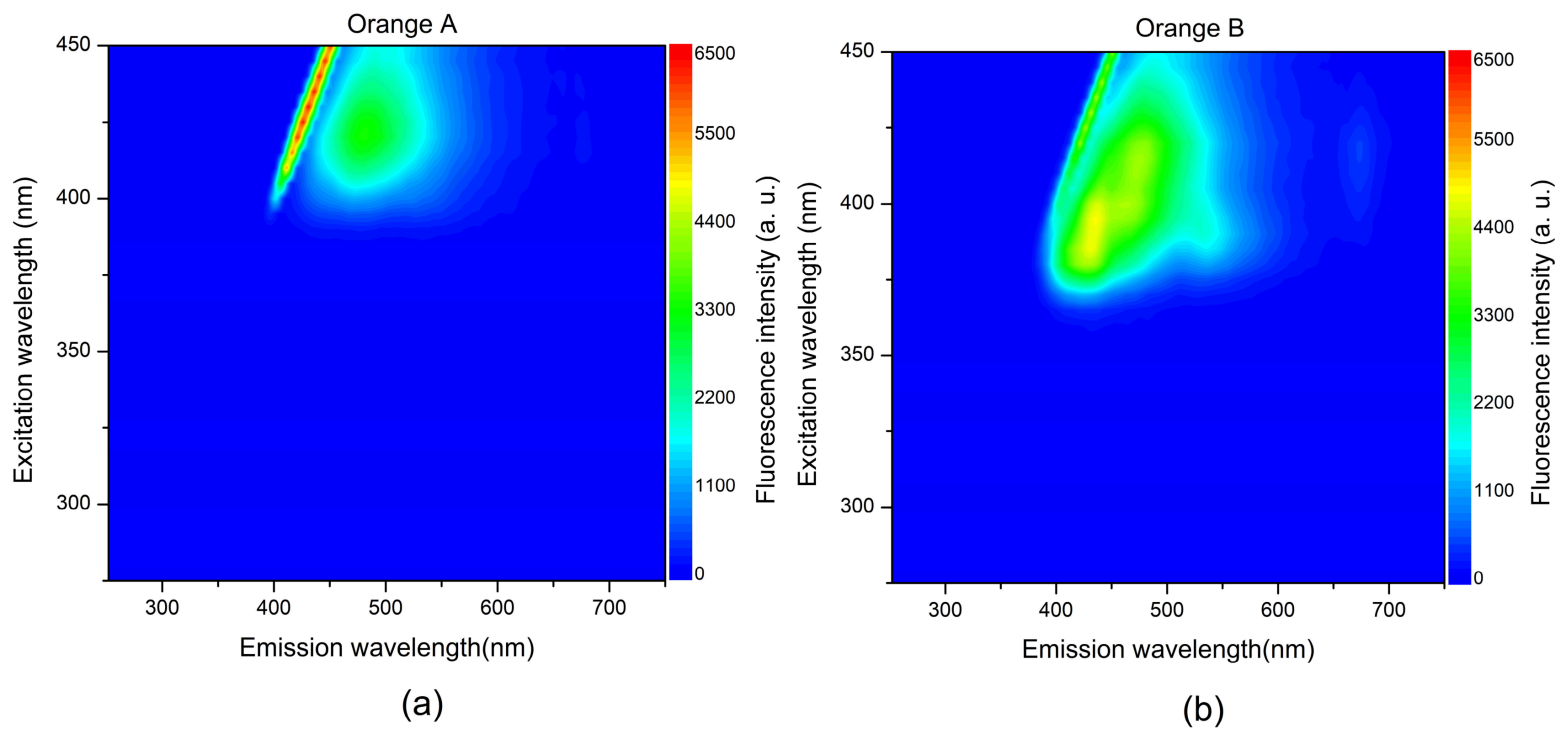
Excitation–emission maps of orange essential oils obtained in the excitation wavelength range of 250–550 nm and emission range of 250–750 nm: (**a**) ORANGE A; (**b**) ORANGE B.

Figure 16a,b show the excitation–emission maps of orange essential oils A and B obtained in the excitation range of 250–550 nm and emission range of 250–750 nm. Orange A exhibited two main fluorescence regions, with excitation bands between approximately 400 and 450 nm and emission centered near 390 nm and between 440 and 540 nm. In contrast, Orange B displayed a broader fluorescence distribution, characterized by excitation wavelengths ranging from approximately 390 to 520 nm and emission extending from about 370 to 580 nm.

**Figure 17:**
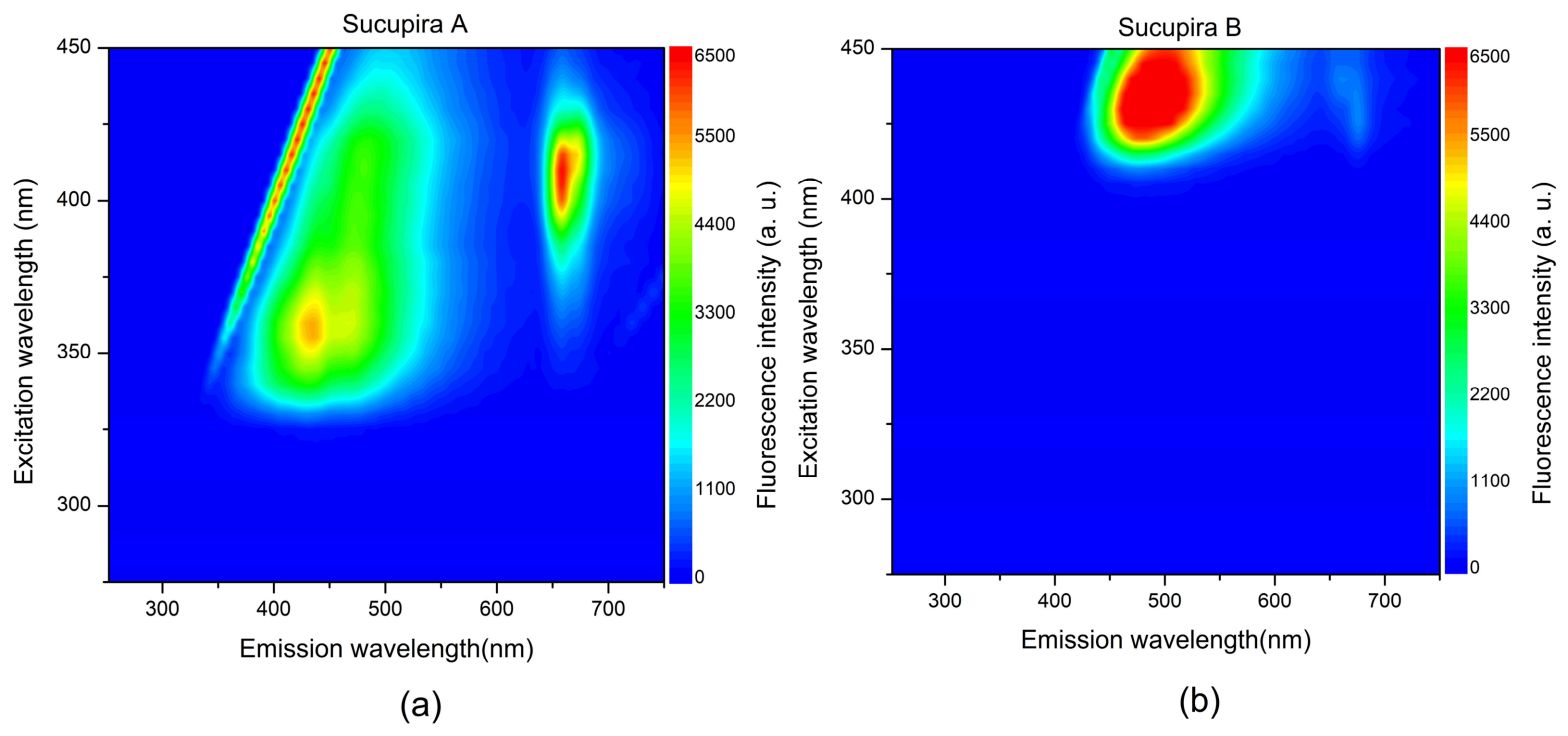
Excitation–emission maps of sucupira essential oils obtained in the excitation wavelength range of 250–550 nm and emission range of 250–750 nm: (**a**) SUCUPIRA A; (**b**) SUCUPIRA B.

Figure 17 presents the excitation–emission maps obtained for sucupira oils A and B within the excitation wavelength range of 250–550 nm and emission interval of 250–750 nm.

According to Figure 17a, sucupira oil A exhibited three main fluorescence regions. The first fluorescence band was observed under excitation wavelengths between approximately 340 and 450 nm, with emission centered near 345 nm. The second and broader fluorescence region corresponded to excitation wavelengths ranging from approximately 330 to 440 nm and emission distributed between 380 and 590 nm. A third fluorescence band was identified at excitation wavelengths between approximately 375 and 430 nm, with emission extending from about 640 to 690 nm.

In contrast, Figure 17b shows that sucupira oil B presented a more concentrated fluorescence profile, characterized by a predominant excitation band between approximately 410 and 450 nm and corresponding emission signals distributed between 445 and 580 nm.

**Figure 18:**
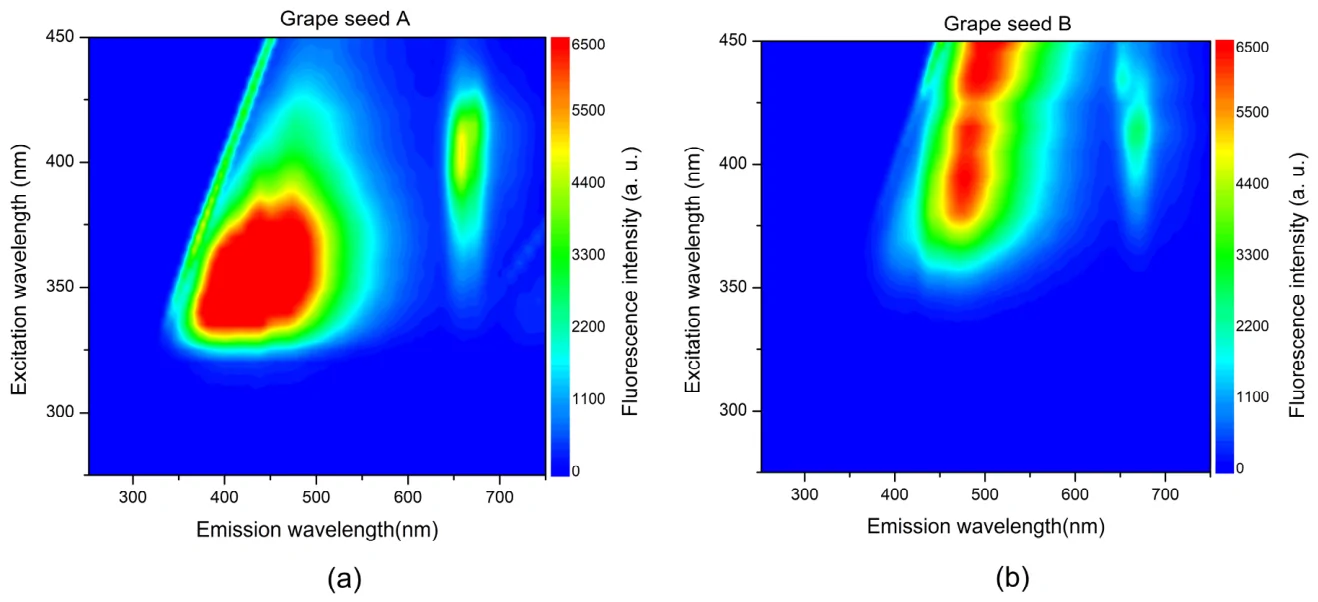
Excitation–emission maps of grape seed oils obtained in the excitation wavelength range of 250–550 nm and emission range of 250–750 nm: (**a**) GRAPE SEED A; (**b**) GRAPE SEED.

According to Figure 18a, grape seed oil A exhibited two main fluorescence regions. The first fluorescence band was observed under excitation wavelengths ranging from approximately 325 to 450 nm, with corresponding emission distributed between 348 and 560 nm. A second fluorescence region was identified at excitation wavelengths between approximately 360 and 425 nm, with emission extending from about 640 to 680 nm.

In contrast, Figure 18b shows the excitation–emission profile of grape seed oil B, which also presented two fluorescence regions. The first band exhibited excitation wavelengths between approximately 360 and 450 nm, with emission distributed from 410 to 590 nm. The second fluorescence region was characterized by excitation wavelengths ranging from approximately 390 to 440 nm and emission between 650 and 690 nm.

## 3. Discussion

### 3.1. DSC Thermograms and Thermal Transition Profiles

The DSC curves obtained for the analyzed oils demonstrated distinct crystallization and melting behaviors, reflecting differences in fatty acid composition, degree of unsaturation, molecular organization, and the presence of minor bioactive constituents such as tocopherols, sterols, phenolic compounds, carotenoids, and volatile terpenes [1,2,3,4,5,6]. Differential scanning calorimetry has been widely applied to investigate phase transitions and thermo-oxidative stability in lipid systems because crystallization and melting events are directly associated with triacylglycerol composition and lipid polymorphism [18,30].

Table 1 summarizes the principal thermal parameters obtained from DSC analyses, including initial temperature, peak max of temperature, and transition enthalpy during cooling and heating cycles. These parameters are important because onset and peak temperatures provide information regarding phase transition behavior, whereas enthalpy values reflect the energy required for crystallization or melting processes, being directly associated with molecular organization, lipid packing efficiency, and thermal stability [18,30].

Among the analyzed samples, avocado oil (Figure 1) exhibited the most complex thermal behavior, presenting two exothermic crystallization events during cooling. The first crystallization transition began at −14.46 °C, with a peak at −20.36 °C and enthalpy of 4.372 J/g, whereas the second transition started at −51.07 °C and reached a peak at −58.42 °C, with enthalpy of 13.290 J/g. During heating, a single endothermic melting event was observed, with onset at −30.56 °C and peak temperature at −8.83 °C, associated with elevated enthalpy (59.15 J/g). The presence of multiple crystallization events suggests heterogeneous triacylglycerol fractions crystallizing at different temperatures, which is characteristic of oils rich in monounsaturated fatty acids and structurally diverse lipid fractions [6,23,28].

Previous studies have described avocado oil as predominantly composed of oleic acid, accompanied by palmitic and linoleic acids [6,23]. Flores et al. [6] reported that avocado oil presents elevated oxidative and thermal stability due to its high MUFA content and antioxidant compounds such as tocopherols and carotenoids. Salgado et al. [23] demonstrated characteristic polymorphic melting profiles associated with triacylglycerol organization, while Chaiyasut et al. [28] reported that extraction processes directly influence avocado oil physicochemical and thermal properties. Aktar and Adal [29] additionally associated avocado oil oxidative resistance with fatty acid composition and activation energy under Rancimat conditions. Wang et al. [34] further demonstrated that chromatographic fatty acid profiling is an effective approach for authentication and quality evaluation of avocado oils. 

In Figure 2, DSC curves show the crystallization (upper curve) and melting behavior (lower curve) of eucalyptus oil samples. Both eucalyptus oil samples (Figure 2) exhibited a single crystallization event during cooling and a single melting event during heating, indicating a straightforward thermal profile. Essential oils are predominantly composed of volatile terpenoids and oxygenated compounds rather than triacylglycerols, explaining the lower complexity of thermal transitions compared with fixed vegetable oils [3,26]. Elaissi et al. [8] reported that eucalyptus essential oil contains elevated concentrations of monoterpenes, especially 1,8-cineole, which strongly influence volatility and thermal behavior. 

In contrast, the orange essential oil samples (Figure 3) did not exhibit any detectable crystallization or melting transitions within the evaluated temperature range of 50 to − 60 °C, under a cooling/heating rate of 20 °C min^−1^ and a nitrogen atmosphere (50 mL min^−1^). This lack of thermal events indicates that the major volatile constituents of the orange oil remained in the liquid state, without undergoing first-order phase transitions even at sub-zero temperatures up to −60 °C. This behavior may be associated with the predominance of limonene and other volatile monoterpenes presenting low molecular organization and reduced crystallization capacity [9,10,26]. Geraci et al. [9] reported that limonene represents most of the chemical composition of citrus essential oils, contributing to high volatility and reduced thermal stability. Razola-Díaz et al. [10] also demonstrated that citrus oils are highly susceptible to volatilization and oxidation processes because unsaturated terpene structures hinder the formation of stable crystalline phases detectable by DSC.

Differential Scanning Calorimetry (DSC) curves show the crystallization (upper curve) and melting behavior (lower curve) of sucupira oil samples are presented in Figure 4. The curves obtained for the sucupira oil samples (Figure 4) revealed distinct thermal profiles, reflecting clear compositional variations between sucupira oil A and sucupira oil B. Ferreira et al. [11] reported that sucupira oils contain biologically active terpenoid compounds capable of influencing lipid organization and thermal stability. Costa-Singh and Jorge [12] additionally described antioxidant and anti-inflammatory activities associated with these constituents. Torres et al. [25] demonstrated that *Pterodon emarginatus* essential oil exhibits complex DSC profiles associated with sesquiterpenes, terpenoids, and fatty derivatives. Given that both commercial samples were obtained via cold-pressing, the observed thermal discrepancies between sucupira oils A and B cannot be attributed to the extraction method itself. Instead, these variations (Figure 4) are likely associated with specific processing parameters, the geographic origin of the seeds, storage conditions, or distinct profiles of secondary metabolites and minor lipid components.

The grape seed oils (Figure 5) exhibited thermal profiles characterized predominantly by single crystallization and melting events. Fernandes et al. [5] reported that cold-pressed grape seed oils generally contain elevated concentrations of linoleic acid and PUFA, directly influencing oxidative susceptibility and thermal behavior. Rockenbach et al. [13] and Garavaglia et al. [14] described grape seed oil as highly enriched in linoleic acid and antioxidant compounds, whereas Bail et al. [24] associated oxidative stability with phenolic compounds and fatty acid composition. The lower crystallization temperatures observed for grape seed oils in the present study are therefore consistent with their elevated PUFA content and reduced molecular packing efficiency [14,30].

Overall, the DSC results demonstrated that the thermal behavior of the analyzed oils is strongly influenced by lipid composition, molecular organization, extraction conditions, and chemical complexity [2,15,18]. Oils richer in monounsaturated fatty acids, such as avocado oil, exhibited greater thermal organization and higher enthalpy values, whereas essential oils rich in volatile terpenes presented simpler or poorly defined transitions [3,26]. Additionally, the presence of multiple crystallization or melting events indicates heterogeneous triacylglycerol distributions and polymorphic behavior, important parameters for evaluating physicochemical stability and oxidative resistance [18,30].

Similar DSC parameters, including initial temperature, peak temperature, and transition enthalpy, have been previously reported for edible oils and lipid systems [18,23,28,29,30]. However, most previous investigations focused exclusively on isolated thermal analyses without integrating chromatographic, oxidative, and spectroscopic approaches. In the present study, the simultaneous interpretation of DSC thermodynamic parameters together with fatty acid composition, oxidative induction time, UV–Vis absorption, and fluorescence spectroscopy provided a broader understanding of the relationship between molecular organization and thermo-oxidative stability in both vegetable and essential oils.

### 3.2. Fatty Acid Composition Determined by Gas Chromatography

The fatty acid composition obtained by gas chromatography (Table 2) demonstrated marked differences among the analyzed oils, reflecting distinct physicochemical properties, thermo-oxidative behaviors, and potential biological applications. Table 2 provides a comparative overview of saturated fatty acids (SFA), monounsaturated fatty acids (MUFA), and polyunsaturated fatty acids (PUFA), allowing direct correlation between lipid composition and the thermal, oxidative, and spectroscopic behavior observed by DSC, Rancimat, UV–Vis, and fluorescence analyses. In general, avocado oil showed predominance of MUFA, grape seed and sucupira oils were characterized by elevated PUFA concentrations, whereas eucalyptus and sweet orange oils exhibited profiles dominated by volatile short-chain compounds.

Avocado oil exhibited predominance of MUFA (63.15%), mainly associated with oleic acid (C18:1n9c), which represented 60.63% of the total fatty acid composition (Table 2). Among saturated fatty acids, palmitic acid (C16:0) was the major constituent, corresponding to 7.35%, while PUFA represented 20.16%, mainly linoleic acid (15.96%) and α-linolenic acid (3.37%). These results are consistent with those reported by Flores et al. [6], who described avocado oil as containing elevated concentrations of oleic acid together with moderate proportions of palmitic and linoleic acids. Wang et al. [7] additionally demonstrated that avocado oils contain carotenoids, tocopherols, and chlorophyll pigments that contribute significantly to antioxidant protection and oxidative stability. Salgado et al. [23] also observed oleic acid values close to 62%, with MUFA representing approximately 65% of total fatty acids, while Chaiyasut et al. [28] demonstrated that extraction processes directly influence avocado oil physicochemical characteristics. Aktar and Adal [29] further associated oxidative resistance with fatty acid composition under accelerated oxidation conditions. Wang et al. [34] reported that oleic acid was the major fatty acid (60%) in authentic avocado oils evaluated by chromatographic methods. 

The predominance of oleic acid observed in the present study is directly associated with the superior thermal and oxidative stability identified in DSC and Rancimat analyses. Oils rich in MUFAs are generally less susceptible to lipid peroxidation because monounsaturated chains contain fewer reactive double bonds than PUFA-rich systems. This behavior corroborates the higher enthalpy values and greater thermal organization observed in the DSC thermograms of avocado oil.

The eucalyptus and sweet orange oils exhibited unusual chromatographic profiles compared with conventional edible vegetable oils, with saturated compounds representing 99.97% and 99.02% of total detected constituents, respectively (Table 2). In eucalyptus oil, caproic acid (C6:0) was the predominant constituent, whereas sweet orange oil also presented predominance of short-chain volatile compounds and only trace amounts of unsaturated constituents. Although these values differ substantially from conventional fixed oils, they reinforce the highly volatile chemical nature of essential oils.

It is important to emphasize that essential oils are chemically dominated by volatile terpenoid compounds rather than conventional long-chain triglycerides [3,4]. Therefore, the high proportion of short-chain saturated compounds (e.g., C6:0) detected by GC-FID represents a methodological artifact of applying standard transesterification to volatile matrices, rather than a true fatty acid profile. Elaissi et al. [8] reported that eucalyptus essential oil is chemically dominated by oxygenated monoterpenes, particularly 1,8-cineole, which may represent most of the volatile composition depending on species and environmental conditions. Guerrero et al. [27] additionally demonstrated that eucalyptus oils exhibit elevated volatility and narrow thermal transition ranges due to their relatively homogeneous terpene composition.

Geraci et al. [9] demonstrated that limonene represents the major constituent of sweet orange essential oil, while oxygenated derivatives such as linalool and citral occur in smaller concentrations. Razola-Díaz et al. [10] further reported that limonene concentrations may vary substantially according to extraction methodology and storage conditions. Therefore, the chromatographic profile observed in the present study reinforces the distinct chemical behavior of essential oils compared with fixed oils such as avocado and grape seed oils. This volatile composition also explains the reduced thermal complexity observed in DSC curves, characterized by fewer thermal transitions and lower enthalpy values, as well as the extremely low oxidative stability identified by the Rancimat method.

Sucupira oils presented intermediate characteristics between saturated and unsaturated lipid systems, with a predominance of PUFA. Sucupira A showed 51.24% PUFA and 26.24% MUFA, while Sucupira B exhibited an even higher PUFA content (59.37%) and a slightly lower MUFA concentration (25.61%). In both samples, linoleic acid (C18:2n6c) was the major fatty acid. Another relevant observation was the detection of elaidic acid (C18:1n9t), identified by matching retention times with the certified Supelco 37 Component FAME standard. While the relative peak area indicated an elevated presence of C18:1n9t, minor co-elution with closely eluting monoenoic isomers cannot be completely excluded under conventional 30 m polyethylene glycol column separation. Nevertheless, this profile highlights a lipid composition rich in unsaturated fatty acids that may contribute to the biological activities commonly attributed to sucupira oils.

Ferreira et al. [11] identified important diterpenes and vouacapan derivatives in *Pterodon emarginatus* oil together with relevant concentrations of oleic and linoleic acids. Costa-Singh and Jorge [12] reported elevated PUFA concentrations in sucupira oils, with linoleic acid representing a substantial proportion of total fatty acids. Torres et al. [25] also described high contents of bioactive volatile fractions in *Pterodon* oils associated with antioxidant and ethnopharmacological activity. According to these authors, variations in fatty acid composition may occur due to geographical origin, climate conditions, maturation stage, and extraction procedures. The elevated PUFA levels observed in the present study may therefore explain the lower oxidative stability identified for sucupira oils during induction time analyses, since polyunsaturated lipids are highly susceptible to free radical propagation reactions [15].

The grape seed oils exhibited the highest PUFA concentrations among all analyzed samples. Grape seed A contained 56.63% PUFA and 28.36% MUFA, whereas grape seed B presented the highest PUFA content of the study (69.85%) together with the lowest SFA content. Linoleic acid was the predominant fatty acid in both samples. Fernandes et al. [5] demonstrated that cold-pressed grape seed oils produced in different Brazilian regions present substantial compositional variation depending on cultivar and extraction conditions. Rockenbach et al. [13] reported linoleic acid concentrations ranging from approximately 60% to 75% in grape seed oils obtained from Vitis cultivars. Garavaglia et al. [14] also described grape seed oil as one of the richest dietary sources of omega-6 fatty acids, especially linoleic acid. Bail et al. [24] similarly reported elevated linoleic acid concentrations together with important antioxidant compounds such as tocopherols and phenolic substances.

The compositional differences observed between grape seed oils A and B suggest variations associated with grape variety, seed maturity, extraction process, or storage conditions. Additionally, grape seed A presented a higher proportion of elaidic acid than grape seed B, which may indicate differences in lipid isomerization during industrial processing or oxidative degradation. Maszewska et al. [30] demonstrated that oils rich in linoleic acid generally exhibit lower oxidative stability because bis-allylic methylene groups accelerate hydroperoxide formation during oxidation reactions. Similarly, Anwar et al. [33] demonstrated that increasing PUFA concentration significantly reduces oxidative stability in vegetable oils subjected to thermal stress conditions. These results indicate differences in unsaturation degree, lipid organization, and oxidative condition between samples.

Although both avocado and grape seed oils contained elevated concentrations of unsaturated fatty acids, their oxidative behaviors were markedly different. Avocado oil, rich in oleic acid (MUFA), exhibited substantially greater oxidative stability than grape seed oils rich in linoleic acid (PUFA), demonstrating the critical influence of unsaturation degree on thermo-oxidative resistance. This behavior reinforces the direct relationship between molecular structure, lipid organization, and oxidative susceptibility observed throughout the present study.

Overall, the chromatographic results demonstrated that fatty acid composition plays a central role in determining the physicochemical, thermal, spectroscopic, and oxidative properties of vegetable and essential oils. Oils richer in MUFA, particularly avocado oil, exhibited greater thermal organization and oxidative resistance, whereas PUFA-rich oils such as grape seed and sucupira oils showed higher susceptibility to oxidation and lower induction periods. Essential oils displayed distinct volatile and terpene-dominated profiles associated with reduced thermal complexity and rapid oxidative degradation. The strong agreement observed between GC, DSC, Rancimat, UV–Vis, and fluorescence analyses reinforces the importance of integrated multianalytical approaches for comprehensive characterization of complex lipid systems.

Although chromatographic characterization of avocado, grape seed, and sucupira oils has been previously reported [5,23,24,25,34], most investigations focused primarily on fatty acid identification or authenticity assessment. Comparative studies integrating chromatographic composition with thermal transitions, oxidative induction periods, UV–Vis absorption, and fluorescence spectroscopy under identical analytical conditions remain limited, particularly for simultaneous evaluation of fixed vegetable oils and essential oils. Therefore, the present study provides a broader interpretation of the relationship between lipid composition and thermo-oxidative stability in chemically distinct oil systems.

### 3.3. Oxidative Stability and Induction Period Behavior

The oxidative stability index of the analyzed oils, expressed as induction period time, is presented in Table 3, while the conductivity profiles as a function of oxidation time are shown in Figure 6, Figure 7, Figure 8, Figure 9, Figure 10, Figure 11 and Figure 12. Significant differences were observed among the analyzed samples, indicating that fatty acid composition, degree of unsaturation, and the presence of volatile compounds strongly influenced oxidative resistance.

Avocado oil (Figure 6) exhibited the highest induction time among all samples, with a mean value of 12.38 h at 110 °C (Table 3), demonstrating superior oxidative stability compared with the other analyzed oils. This behavior can be directly associated with its elevated monounsaturated fatty acid content (63.15%), particularly oleic acid (60.63%) (Table 2). Flores et al. [6] described avocado oil as one of the most oxidatively stable fruit oils due to its elevated oleic acid concentration and natural antioxidant constituents such as tocopherols, carotenoids, and phytosterols. Wang et al. [7] additionally demonstrated the presence of antioxidant pigments and bioactive compounds that contribute to oxidative protection. Salgado et al. [23] also reported elevated oxidative resistance in avocado oils rich in MUFA, while Aktar and Adal [29] demonstrated that avocado oils rich in oleic acid generally exhibit induction periods ranging from approximately 8 to 15 h under Rancimat conditions, values consistent with those obtained in the present study.

The elevated oxidative stability observed for avocado oil is also consistent with the DSC results (Figure 1), in which this sample exhibited more complex crystallization behavior and greater thermal resistance than the highly polyunsaturated oils. Oils rich in MUFA are generally less susceptible to lipid peroxidation because monounsaturated chains contain fewer reactive double bonds than PUFA-rich systems [15].

The grape seed oil A sample (Figure 11) presented intermediate oxidative stability, with an induction time of 6.18 h. Although lower than avocado oil (12.31 h, Figure 6 and Table 3), this value was substantially higher than those observed for grape seed oil B and the essential oils. The oxidative behavior of grape seed oil A is associated with its fatty acid profile, characterized by elevated PUFA concentration and predominance of linoleic acid (Table 2). Fernandes et al. [5] demonstrated that grape seed oils produced under different extraction conditions may present substantial variations in fatty acid composition and antioxidant content, directly influencing oxidative stability. Rockenbach et al. [13] also reported that grape seed oils commonly contain high linoleic acid concentrations, which contribute to nutritional value but simultaneously reduce oxidative stability. Garavaglia et al. [14] further emphasized that the high degree of unsaturation in grape seed oils increases susceptibility to lipid peroxidation. Bail et al. [24] nevertheless demonstrated that tocopherols and phenolic compounds naturally present in grape seed oils may partially delay oxidation reactions.

In contrast, grape seed oil B (Figure 12) exhibited a dramatically lower induction time (0.04 h, Table 3), despite presenting the highest PUFA content among all analyzed oils and the highest linoleic acid concentration (Table 2). This result demonstrates the strong influence of polyunsaturation on oxidative degradation. Maszewska et al. [30] demonstrated that oils rich in linoleic acid are highly susceptible to oxidation because bis-allylic methylene groups facilitate hydrogen abstraction and hydroperoxide formation. Similarly, Anwar et al. [33] reported that increasing PUFA concentration significantly reduces oxidative stability in vegetable oils subjected to thermal stress conditions. The pronounced difference between grape seed oils A and B suggests that factors such as extraction method, processing conditions, storage, or antioxidant content may also have influenced oxidative stability.

The essential oils of eucalyptus (Figure 7) and sweet orange (Figure 8) exhibited extremely low induction times, corresponding to 0.28 h and 0.02 h (Table 3), respectively. These results indicate very rapid oxidative degradation under accelerated conditions. Unlike fixed vegetable oils, essential oils are predominantly composed of volatile terpenoids rather than long-chain triglycerides [3,4]. Elaissi et al. [8] demonstrated that eucalyptus essential oil is rich in oxygenated monoterpenes such as 1,8-cineole, compounds characterized by elevated volatility and oxidative instability. Geraci et al. [9] and Razola-Díaz et al. [10] similarly reported that citrus essential oils rich in limonene are highly susceptible to oxidation because monoterpenes rapidly form hydroperoxides and oxygenated derivatives upon exposure to heat and oxygen. Turek and Stintzing [26] further emphasized that essential oils rich in volatile short-chain compounds exhibit oxidative behaviors substantially different from those observed for edible vegetable oils. Guerrero et al. [27] additionally demonstrated that eucalyptus essential oil undergoes rapid thermal degradation due to the instability of volatile terpene constituents. Therefore, despite the predominance of saturated compounds identified in Table 2, the oxidative behavior of these oils is governed primarily by terpene oxidation mechanisms rather than conventional fatty acid oxidation pathways.

Sucupira oil A (Figure 9) showed an induction time of 0.02 h, whereas sucupira oil B (Figure 10) exhibited an induction time of 0.01 h, representing the lowest oxidative stability among all analyzed samples (Table 3). These reduced values are directly related to the elevated PUFA concentrations observed in both oils, especially linoleic acid (Table 2). Choe and Min [15] demonstrated that oxidation rates increase proportionally with the number of double bonds present in fatty acids, making PUFA-rich oils more susceptible to oxidative degradation. Ferreira et al. [11] identified diterpenes and vouacapan derivatives in *Pterodon emarginatus* oil together with relevant concentrations of unsaturated fatty acids. Costa-Singh and Jorge [12] also reported elevated concentrations of PUFA in sucupira oils and highlighted their susceptibility to oxidative processes. Torres et al. [25] further described lipid and terpene fractions associated with antioxidant and anti-inflammatory activities in *Pterodon* oils. However, despite the presence of bioactive diterpenes and phenolic compounds, these constituents were insufficient to prevent rapid oxidation under accelerated Rancimat conditions at 110 °C.

Overall, the oxidative stability results demonstrated a clear relationship between induction period and fatty acid composition. Oils rich in monounsaturated fatty acids, particularly avocado oil, exhibited greater oxidative resistance, whereas oils rich in polyunsaturated fatty acids, such as grape seed and sucupira oils, showed reduced induction periods. Essential oils displayed extremely low oxidative stability due to the rapid degradation of volatile terpene compounds. These findings are consistent with the DSC thermal analyses and reinforce the importance of lipid composition, unsaturation degree, and natural antioxidant constituents in determining the oxidative behavior of vegetable and essential oils.

### 3.4. UV–Vis Absorption Characteristics of Vegetable and Essential Oils

Figure 13 presents the UV–Vis absorption spectra obtained for the seven analyzed oil samples, including avocado oil, grape seed oils (A and B), and the essential oils of eucalyptus (A and B), orange (A and B), and sucupira (A and B). The spectral profiles revealed marked differences among the samples, indicating variations in chemical composition, degree of unsaturation, pigment content, and presence of bioactive compounds.

The essential oils of eucalyptus, orange, and sucupira A exhibited absorption bands predominantly in the ultraviolet region, especially between approximately 225 and 280 nm. These absorption regions are commonly associated with electronic transitions of conjugated double bonds, terpenoids, phenolic compounds, and other volatile constituents present in essential oils. According to Elaissi et al. [8], eucalyptus essential oil contains high concentrations of monoterpenes, particularly 1,8-cineole, which strongly absorb in the UV region. Similarly, Geraci et al. [9] and Razola-Díaz et al. [10] demonstrated that citrus essential oils are rich in limonene and oxygenated terpenes, which also contribute to absorption bands at lower wavelengths.

In the present study, eucalyptus and orange oils showed almost complete predominance of saturated compounds, corresponding to 99.97% and 99.02%, respectively, with major contributions from caproic acid (C6:0). The low degree of unsaturation observed by gas chromatography explains the absence of intense absorption bands in the visible region. Furthermore, the low oxidative stability determined by the Rancimat method (0.28 h for eucalyptus and 0.02 h for orange oil) suggests that these oils are highly volatile and thermally unstable.

Sucupira A oil also presented absorption bands in the UV region, indicating the presence of unsaturated compounds and phenolic constituents. According to Ferreira et al. [11], oils from *Pterodon* species contain diterpenes and flavonoids with recognized biological activity, which may contribute to absorption in this spectral region. Likewise, Costa-Singh and Jorge [12] reported antioxidant compounds in sucupira oils capable of influencing spectroscopic behavior. In contrast, Sucupira B did not exhibit well-defined absorption bands, suggesting possible differences in extraction conditions, oxidation level, or concentration of chromophoric compounds between the two samples.

Among all analyzed samples, avocado oil exhibited the most intense and broad absorption bands in the visible region, extending approximately from 410 to 670 nm. This spectral behavior is characteristic of oils rich in chlorophylls, carotenoids, tocopherols, and other natural pigments. According to Flores et al. [6], avocado oil contains considerable concentrations of chlorophyll derivatives and carotenoids, compounds responsible for absorption in the visible region and directly associated with antioxidant activity. The chromatographic analysis performed in this study demonstrated that avocado oil presented the highest concentration of monounsaturated fatty acids (63.15%), especially oleic acid (60.63%), together with relevant levels of linoleic acid (15.96%) and α-linolenic acid (3.37%).

The absorption features observed in the visible region are consistent with the contribution of chromophoric compounds such as carotenoids and chlorophylls, which contribute to the characteristic absorption behavior of vegetable oils [7,22]. In particular, the bands observed between approximately 410 and 670 nm in avocado oil are compatible with the contribution of these natural pigments. In the ultraviolet region, the less intense bands observed around 225 and 265 nm may be associated with electronic transitions involving different chromophoric groups. The absorption near 225 nm can be attributed to π → π* transitions associated with unsaturated or conjugated systems, whereas the band around 265 nm may be related to n → π* transitions involving carbonyl-containing chromophores [21,22].

The strong visible absorption profile observed for avocado oil is also consistent with its elevated oxidative stability (12.38 h), indicating preservation of antioxidant pigments and unsaturated lipid structures. Similar UV–Vis spectral behavior was described by Melo et al. [15], who demonstrated that the presence of carotenoids and tocopherols significantly contributes to absorption between 400 and 500 nm, serving as important indicators of oil quality and oxidative stability.

Grape seed oil A also showed intense absorption bands in the visible region, ranging approximately from 420 to 648 nm, together with less intense bands between 225 and 265 nm. The absorption in the UV region is associated with unsaturated fatty acids and phenolic compounds, while the visible bands may be related to residual pigments and oxidation products. According to Garavaglia et al. [14], grape seed oils contain phenolic compounds and tocopherols with antioxidant activity capable of influencing the UV–Vis spectral profile.

The fatty acid composition of grape seed oil A revealed elevated concentrations of linoleic acid (50.46%) and total polyunsaturated fatty acids (56.63%), which explains its susceptibility to oxidation despite the presence of antioxidant compounds. Nevertheless, grape seed oil A exhibited relatively higher oxidative stability (6.18 h) compared with grape seed oil B.

In contrast, grape seed oil B did not present expressive absorption bands in either the UV or visible regions. This behavior may indicate degradation of pigments and antioxidant molecules due to oxidation processes. This interpretation agrees with its Rancimat induction time of only 0.04 h, demonstrating low oxidative stability. Additionally, grape seed oil B presented the highest PUFA concentration among all analyzed samples (69.85%), particularly linoleic acid (66.84%), which increases susceptibility to lipid oxidation. Similar compositional variations among grape seed oils from different origins were also discussed by Fernandes et al. [5].

Overall, the UV–Vis absorption spectra demonstrated strong correlation with chromatographic composition and oxidative stability analyses. Oils richer in natural antioxidants, pigments, and monounsaturated fatty acids, such as avocado oil, exhibited broader and more intense absorption bands, whereas oils characterized by high volatility or elevated PUFA content showed reduced spectral complexity and lower oxidative stability. The results reinforce the applicability of UV–Vis spectroscopy as a rapid and efficient analytical tool for evaluating oil composition, oxidation state, and quality [5,6,14,15].

### 3.5. Excitation–Emission Fluorescence Features of the Oils

The excitation–emission fluorescence matrices obtained for the vegetable and essential oils revealed distinct spectral profiles among the analyzed samples (Figure 14, Figure 15, Figure 16, Figure 17 and Figure 18), demonstrating differences in chemical composition, degree of unsaturation, and presence of natural fluorophores such as tocopherols, chlorophyll derivatives, carotenoids, phenolic compounds, and oxidation products.

The avocado oil fluorescence map (Figure 14) exhibited intense emission regions distributed mainly between 300 and 550 nm, with excitation wavelengths centered between 270 and 420 nm. This behavior is characteristic of oils rich in unsaturated fatty acids and natural antioxidant compounds, especially tocopherols and chlorophyll pigments. According to Flores et al. [6], avocado oil contains considerable concentrations of chlorophylls, carotenoids, tocopherols, and phytosterols, compounds directly associated with fluorescence emission in the visible region. These constituents may explain the broader fluorescence regions observed for avocado oil compared with the other samples.

In the present study, the intense fluorescence observed for avocado oil is consistent with its elevated content of oleic acid (60.63%), linoleic acid (15.96%), and α-linolenic acid (3.37%), as determined by gas chromatography. Additionally, the higher oxidative stability obtained by the Rancimat method (12.38 h) indicates that antioxidant compounds naturally present in the oil contributed to reducing oxidative degradation and preserving fluorescent molecules. Similar fluorescence behavior in oils rich in pigments and antioxidant compounds was described by Melo et al. [15].

The eucalyptus essential oil (Figure 15) presented low fluorescence intensity, with emission concentrated predominantly in the ultraviolet region. This behavior is associated with its chemical composition characterized mainly by volatile terpenoids and short-chain saturated compounds. According to Elaissi et al. [8], eucalyptus essential oil is rich in monoterpenes such as 1,8-cineole, compounds that exhibit weaker fluorescence signals compared with chlorophylls and polyunsaturated lipid compounds. The GC results demonstrated that eucalyptus oil contained 99.97% saturated compounds, especially caproic acid (86.72%) and butyric acid (13.23%), which explains the absence of broader fluorescence bands. Moreover, the low oxidative stability observed by the Rancimat test (0.28 h) reinforces the high volatility and susceptibility of this oil to thermal degradation.

Similarly, the orange essential oil (Figure 16) exhibited weak fluorescence intensity concentrated mainly in the ultraviolet region. Citrus essential oils are composed predominantly of limonene and other volatile terpenes, which generally present limited fluorescence responses due to their molecular structure, as previously described by Geraci et al. [9] and Razola-Díaz et al. [10]. The chromatographic analysis showed that orange oil was composed almost entirely of saturated compounds (99.02%), especially caproic acid (93.64%), with minimal concentrations of MUFA and PUFA (<1%). The extremely low induction period (0.02 h) indicates low oxidative resistance and instability under heating conditions, corroborating the reduced fluorescence profile observed experimentally.

The fluorescence spectra obtained for Sucupira oils A and B (Figure 17a,b) demonstrated moderate fluorescence regions, although differences between the samples were evident. Sucupira A presented broader and more intense fluorescence regions than Sucupira B, suggesting differences in the concentration of fluorescent bioactive compounds or distinct oxidative conditions between samples. Both oils exhibited elevated concentrations of polyunsaturated fatty acids, particularly linoleic acid, corresponding to 45.30% in Sucupira A and 52.57% in Sucupira B. Additionally, the presence of elaidic acid was considerable in both oils, with values of 24.27% and 23.66%, respectively.

According to Ferreira et al. [11] and Costa-Singh and Jorge. [12], species of the genus *Pterodon* contain diterpenes, flavonoids, and phenolic constituents with recognized antioxidant and antimicrobial activities. These compounds may contribute to fluorescence emission in the visible region. However, despite the elevated PUFA content, both sucupira oils showed very low oxidative stability (0.02 h for Sucupira A and 0.01 h for Sucupira B), indicating rapid oxidative degradation, which may reduce fluorescence intensity due to degradation of native fluorophores and formation of secondary oxidation products.

The grape seed oils (Figure 18a,b) also exhibited distinct fluorescence behaviors between samples A and B. Grape seed oil A presented broader and more intense fluorescence bands, whereas grape seed oil B exhibited markedly lower fluorescence intensity. This behavior is strongly associated with the fatty acid composition and oxidative stability of the samples. Grape seed oil B showed the highest concentration of polyunsaturated fatty acids among all analyzed oils (69.85%), mainly linoleic acid (66.84%), while grape seed oil A presented 56.63% PUFA and 50.46% linoleic acid.

The excitation–emission fluorescence maps and oxidative stability results demonstrate that grape seed oils from the same botanical species (*Vitis vinifera*) may exhibit substantial differences in their physicochemical and thermal behavior depending on the commercial source, processing conditions, and composition. In the present study, the oils identified as grape seed oil A and grape seed oil B were produced by different Brazilian companies. Grape seed oil A was manufactured by Aroma D’Alma (Cambé, PR, Brazil), whereas grape seed oil B was produced by WNF Óleos Essenciais (São Paulo, SP, Brazil). Both products were obtained by cold pressing and derived from the same species (*Vitis vinifera*). In contrast, Fernandes et al. [5] also evaluated grape seed oil from *Vitis vinifera*; however, the samples were acquired from another Brazilian manufacturer, Indústria Pazze Alimentos™, Panambi (RS), Brazil. These differences highlight that even oils obtained from the same plant species may present distinct fluorescence profiles and oxidative stability depending on industrial processing, raw material quality, storage conditions, and natural compositional variability.

According to Garavaglia et al. [14] and Fernandes et al. [5], grape seed oils contain important antioxidant compounds such as tocopherols and phenolic substances, which contribute to fluorescence emission and oxidative protection. Nevertheless, oils rich in polyunsaturated fatty acids are generally more susceptible to lipid oxidation [2,15]. This behavior was confirmed in the present study by the Rancimat results, where grape seed oil A presented an induction period (IP) of 6.18 h, while grape seed oil B exhibited a markedly lower IP of only 0.04 h. Fernandes et al. [5], evaluating grape seed oil from another Brazilian manufacturer, reported an induction period of 6.66 h for grape seed oil samples (G1 and G2), demonstrating oxidative stability values close to those observed for grape seed oil A in the present work. The lower fluorescence intensity and reduced oxidative stability observed for grape seed oil B may therefore indicate greater oxidative degradation and depletion of fluorescent antioxidant compounds.

The excitation–emission maps also revealed important differences among the samples. The fluorescence map reported by Fernandes et al. [5] exhibited intense emission regions mainly between 390–450 nm under excitation wavelengths around 300–320 nm, which are commonly associated with tocopherols, phenolic compounds, and oxidation-related fluorophores [20,22,31]. Similarly, grape seed oil A (Figure 18.a) presented an intense fluorescence region concentrated approximately between 400–520 nm emission and 330–390 nm excitation, indicating the presence of fluorescent antioxidant constituents and compounds related to lipid structures. In contrast, grape seed oil B (Figure 18.b) showed broader but less intense fluorescence regions, with maxima displaced toward higher excitation wavelengths (approximately 380–450 nm) and emission between 430–550 nm. These spectral differences suggest modifications in the chemical composition and possible degradation of natural antioxidants, which may be associated with oxidation processes [20,31,32].

The diagonal bands observed in all excitation–emission maps correspond to Rayleigh scattering phenomena, which are characteristic artifacts commonly observed in fluorescence spectroscopy of edible oils [20,31]. Despite not representing real fluorophores, these regions confirm the high sensitivity of the spectroscopic measurements. The observed spectral differences may also be related to factors such as extraction process, storage conditions, raw material quality, and fatty acid composition [23,24,28,34]. Vegetable oils rich in unsaturated fatty acids are more susceptible to oxidation, leading to the formation of fluorescent secondary compounds during degradation [2,15,29,30]. Therefore, the fluorescence profiles obtained in this study reinforce the applicability of excitation–emission spectroscopy as a useful tool for evaluating the chemical characteristics and oxidative stability of grape seed oils. Importantly, as demonstrated across the studied matrices, these excitation–emission fluorescence maps function as a unique optical “fingerprint” characteristic of each oil’s botanical origin, commercial brand, and processing history [20,22]. Because native fluorophores and oxidation products are highly sensitive to chemical changes, total luminescence spectroscopy acts as a rapid, high-throughput diagnostic tool for oil identification, authentication, and quality screening with minimal sample preparation [20,31,32].

Furthermore, the results obtained in this study agree with those previously described for vegetable oils rich in antioxidant pigments and phenolic compounds, in which fluorescence spectroscopy proved to be an efficient technique for evaluating oxidative degradation and distinguishing oils according to chemical composition and antioxidant content. Melo et al. [16] demonstrated that oils with higher oxidative stability and greater concentrations of natural antioxidants exhibited more intense and organized fluorescence profiles, whereas oxidized oils or oils poor in antioxidant compounds presented reduced fluorescence intensity and less defined spectral regions. Similar behavior was observed in the present work, especially for grape seed oil A, which displayed stronger fluorescence signals and greater oxidative stability compared with grape seed oil B.

Furthermore, the fluorescence displacement toward longer emission wavelengths observed in grape seed oil B may indicate the formation of secondary oxidation products, such as aldehydes, ketones, and conjugated compounds generated during lipid oxidation [2,15]. Similar spectral behavior has been described in oxidized edible oils, where fluorescence signals shift due to structural changes in unsaturated fatty acids and degradation of tocopherols [20,31]. Overall, the fluorescence spectroscopy results showed strong agreement with the DSC, GC, UV–Vis, and Rancimat analyses, demonstrating that oils with higher antioxidant preservation and greater oxidative stability exhibited more intense and organized fluorescence profiles, whereas samples with lower oxidative resistance showed weaker fluorescence emissions and broader spectral regions.

## 4. Materials and Methods

### 4.1. Acquisition of Oil Samples

The essential and vegetable oils used in this study were commercially acquired from different Brazilian companies specialized in aromatherapy, natural products, and cold-pressed oils. Information regarding botanical species, extraction method, declared composition, and product origin was obtained from manufacturers’ specifications and complemented with data available in the scientific literature. The comparative evaluation between commercial samples (designated as A and B) was specifically designed to investigate commercial variability, differences in industrial processing, geographical origin, and storage conditions among products derived from the same botanical species.

The avocado oil (*Persea gratissima*) was extracted by cold pressing and manufactured by WNF Óleos Essenciais (São Paulo, SP, Brazil; manufactured in 2023, 36-month shelf life). The eucalyptus essential oil (*Eucalyptus globulus*) was produced by steam distillation of the leaves and commercialized as pure essential oil by By Samia (Itapevi, SP, Brazil; manufactured in 2023, 36-month shelf life) and Harmonie Aromaterapia (Florianópolis, SC, Brazil; manufactured in 2023, 36-month shelf life). For didactic purposes and to facilitate identification throughout the manuscript, these samples were designated as Eucalyptus A and Eucalyptus B, respectively. The sweet orange essential oil (*Citrus aurantium* var. *dulcis*) was extracted from fruit peels by cold pressing and commercialized by By Samia (Itapevi, SP, Brazil; manufactured in 2023, 36-month shelf life) and Beleza do Campo (São Paulo, SP, Brazil; manufactured in 2023, 36-month shelf life), being designated as Orange A and Orange B, respectively.

For grape seed oils (*Vitis vinifera*), two distinct commercial samples obtained by cold pressing were evaluated: sample “Grape Seed A”, manufactured by Aroma D’Alma (Cambé, PR, Brazil; manufactured in 2023, 36-month shelf life), and sample “Grape Seed B”, produced by Beleza do Campo (São Paulo, SP, Brazil; manufactured in 2023, 36-month shelf life). The inclusion of different commercial brands enabled the evaluation of possible compositional and oxidative variations among oils derived from the same botanical species subjected to distinct industrial and commercial conditions.

For sucupira oil (*Pterodon emarginatus*), two distinct commercial samples obtained by cold pressing of the seeds were also analyzed: sample “Sucupira A”, manufactured by Amazon Leve (São Paulo, SP, Brazil; manufactured in 2023, 36-month shelf life), and sample “Sucupira B”, obtained from Herbomel Biomédica e Ambiental LTDA (São Lourenço, MG, Brazil; manufactured in 2023, 36-month shelf life), allowing a comparative evaluation between different commercial sources.

All oil samples were purchased in amber glass bottles and stored unopened at room temperature (22 ± 2 °C), fully protected from light exposure prior to analysis. This standardized condition was controlled because differences in secondary metabolite composition, geographic origin of the seeds, processing conditions, and storage environment may directly influence the thermal and oxidative stability of the oils.

The commercial samples were maintained in their original amber containers and stored under refrigeration until analysis. Approximately 50 mL of avocado oil, 50 mL of grape seed oil sample A, and 100 mL of grape seed oil sample B were used in the experiments, together with six 10 mL bottles of eucalyptus essential oil, six 10 mL bottles of sweet orange essential oil, and sucupira oil samples corresponding to 20 mL of sample A and 40 mL of sample B.

The differences observed among samples derived from the same botanical species were subsequently correlated with the results obtained by Differential Scanning Calorimetry (DSC), Gas Chromatography (GC), UV–Vis spectroscopy, fluorescence spectroscopy, and oxidative stability determination using the Rancimat method.

### 4.2. Thermal Characterization Procedure

Differential Scanning Calorimetry (DSC) curves were obtained for avocado, eucalyptus, grape seed, sucupira, and sweet orange oils using a DSC-Q20 instrument coupled to an RCS90 cooling system (TA Instruments, New Castle, DE, USA). For each oil, the same sample was analyzed three consecutive times under identical experimental conditions to assess the repeatability of the thermal measurements (n = 3 measurements per sample). The calorimeter was calibrated for temperature and heat flow using high-purity indium (Tm = 156.60 °C, ΔH = 28.45 J/g). Approximately 7.0 ± 1.0 mg of each oil sample was weighed into sealed aluminum crucibles, while an empty crucible of the same type was used as the reference.

Measurements were performed under a nitrogen atmosphere with a flow rate of 50 mL min^−1^. Heating and cooling cycles were conducted at a rate of 20 °C min^−1^ within a temperature range from −60 °C to 50 °C. The three repeated measurements for each sample showed no relevant differences in the heating and cooling curves, indicating good repeatability of the thermal profiles. Therefore, a representative curve was used for the presentation and interpretation of the thermal behavior of each oil. Thermal behavior was assessed based on the integration of the peak area using the sig-horizontal tool of the TA Advantage/Universal Analysis software (version 4.5A, TA Instruments, New Castle, DE, USA).

### 4.3. Chromatographic Determination of Fatty Acids

The fatty acid profiles of avocado, eucalyptus, grape seed, sucupira, and sweet orange oils were determined following the extraction procedure described by Bligh and Dyer [35], based on the solvent proportion system involving methanol, chloroform, and water (2:1:0.8, *v*/*v*/*v*). Fatty acid methyl esters (FAMEs) were obtained after alkaline saponification of the samples with methanolic NaOH, followed by esterification using a methanolic solution containing H_2_SO_4_ and NH_4_Cl, according to previously reported methodologies [36,37]. The resulting esters were extracted into hexane prior to chromatographic analysis.

FAME analyses were performed for each oil sample using a gas chromatograph (CP-3800, Varian, Santa Clara, CA, USA) equipped with a flame ionization detector (FID), a split/splitless injector (operated in split mode, split ratio 1:50), and a fused silica capillary column coated with polyethylene glycol stationary phase (Carbowax 20M, 30 m × 0.25 mm ID × 0.25 µm film thickness, Quadrex, Santa Clara, CA, USA). Chromatographic conditions were established as follows: injector and detector temperatures were maintained at 250 °C; initial column temperature of 80 °C held for 2 min, followed by a heating ramp of 4 °C min^−1^ up to 220 °C, with a final holding time of 13 min. Hydrogen was used as the carrier gas at a constant flow rate of 1.0 mL min^−1^, and the injection volume was 1.0 µL.

Compound identification was achieved by strictly comparing chromatographic retention times (t_r_) with those of a certified reference standard mixture analyzed under identical operational conditions (Supelco 37-Component FAME Mix, C4:0–C24:0, including C18:1n9$ and C18:2n6t standards, Sigma-Aldrich, Darmstadt, Germany). Chromatographic conditions provided complete baseline separation for critical isomer pairs, including cis/trans geometric configurations (C18:1n9t at t_r_ ≈18.16–18.22 min vs. C18:1n9c at t_r_ ≈ 18.26–18.29 min, and C18:2n6t vs. C18:2n6c). Quantification was performed by integrating peak areas, applying theoretical FID response correction factors (f_FID_) relative to palmitic acid (C16:0), and expressing the results as corrected relative area percentages (% FAME) and mass yield (mg/g of oil), as detailed in Table 2. To assess analytical repeatability, repeated injections of the same prepared sample were performed under identical chromatographic conditions. The repeated chromatographic analyses showed no relevant differences in retention times (±0.02 min variation), peak profiles, or relative fatty-acid composition, confirming the reproducibility of the analytical procedure. Therefore, the fatty-acid composition reported for each oil corresponds to the analytical determination obtained from the individual sample, while the repeated injections were used solely to verify chromatographic repeatability. It is important to note that this chromatographic analysis was exclusively dedicated to fatty acid methyl ester (FAME) profiling; volatile terpenoid constituents (such as 1,8-cineole or limonene) were not targeted, analyzed, or experimentally identified by GC-FID in this study.

### 4.4. Rancimat Oxidation Assay

The oxidative stability of avocado, eucalyptus, grape seed, sucupira, and sweet orange oils was evaluated using a Rancimat 893 apparatus (Metrohm, Basel, Switzerland) according to the standardized procedure established by European Standard EN 14112 [38]. For each oil sample, 3.0 g was subjected to accelerated oxidation at 110 °C under a continuous airflow of 10 L h^−1^. The oxidation induction period (IP, h) was automatically determined by the instrument software based on the conductivity changes generated during the oxidation process. The reported IP values correspond to individual analytical determinations obtained under identical testing conditions. The standardized EN 14112 protocol was applied across all matrices to enable a direct comparative evaluation between fixed lipid systems and volatile essential oil matrices. Because conductivity-based Rancimat detection does not inherently discriminate between physical evaporation and chemical degradation without hyphenated mass spectrometry, the induction period recorded for terpene-rich essential oils (eucalyptus and sweet orange) reflects the combined effect of rapid thermal volatilization under continuous airflow and thermo-oxidative breakdown of monoterpene constituents [26,27].

### 4.5. UV-Vis Absorbance Spectroscopy

For the optical measurements, vegetable oil samples were diluted in HPLC-grade hexane (99.9%, Sigma-Aldrich, St. Louis, MI, USA) at concentrations of 0.12% and 50% (*v*/*v*). The prepared solutions were analyzed individually using a quartz cuvette with four polished optical faces and a 10 mm optical path length. UV– Vis absorption spectra were recorded using a Lambda 265 UV/Vis spectrophotometer (PerkinElmer, Waltham, MA, USA) equipped with a silicon photodiode array detector over the wavelength range of 200–900 nm, with a fixed spectral bandwidth (slit width) of 1.5 nm and a data interval (spectral step) of 1 nm at room temperature. The same prepared solution was measured repeatedly under identical instrumental conditions to verify spectral repeatability, and no relevant differences were observed among the resulting spectra. The resulting spectra were used to characterize the molecular absorption profiles of the analyzed oil samples under the specified experimental conditions.

### 4.6. Fluorometric Mapping Acquisition

Excitation–emission fluorescence matrices (EEMs) were obtained using an FS-2 bench-top spectrofluorometer (Scinco, Seoul, Republic of Korea). For fluorescence analyses, the oil samples were diluted to 50% (*v*/*v*) in HPLC-grade hexane. The excitation wavelengths ranged from 270 to 450 nm in 10 nm increments, while emission spectra were collected from 250 to 750 nm. Both excitation and emission slit widths were set to 5 nm, and the detector sensitivity was adjusted to 600 V. Measurements were performed using a four-sided quartz cuvette with a 10 mm optical path length. The same prepared solution was measured repeatedly under identical instrumental conditions to verify the repeatability of the fluorescence response, and no relevant differences were observed among the resulting excitation–emission fluorescence matrices. The resulting EEMs were used to characterize the fluorescence profiles of the analyzed oil samples under the specified experimental conditions.

## 5. Conclusions

The present study demonstrated that the combined application of differential scanning calorimetry (DSC), gas chromatography (GC), Rancimat oxidative stability analysis, UV–Vis absorption spectroscopy, and fluorescence spectroscopy provides a comprehensive and complementary approach for evaluating the physicochemical and thermo-oxidative properties of vegetable and essential oils.

The results revealed that fatty acid composition was the main factor governing thermal behavior and oxidative stability. Avocado oil exhibited the highest thermo-oxidative stability due to its elevated monounsaturated fatty acid content, especially oleic acid, together with the presence of antioxidant compounds such as carotenoids, chlorophyll derivatives, and tocopherols. This composition resulted in greater molecular organization, higher transition enthalpies, broader spectroscopic profiles, and the longest induction period among all analyzed samples.

In contrast, grape seed and sucupira oils showed higher concentrations of polyunsaturated fatty acids, particularly linoleic acid, which were directly associated with lower oxidative stability and greater susceptibility to lipid peroxidation. Differences observed between samples of the same oil type, especially grape seed oils A and B and sucupira oils A and B, demonstrated that extraction conditions, storage, oxidation level, and compositional variability strongly influence physicochemical behavior and oxidative resistance.

The essential oils of eucalyptus and orange exhibited distinct behavior compared with fixed vegetable oils due to their volatile terpene-rich composition. These oils presented simpler thermal transitions, weak spectroscopic responses in the visible region, and extremely low induction periods, indicating high thermal instability and rapid oxidative degradation under accelerated conditions.

The strong agreement observed among DSC, GC, Rancimat, UV–Vis, and fluorescence analyses confirmed the effectiveness of integrated multianalytical approaches for characterizing complex lipid systems. Furthermore, the study demonstrated that spectroscopic and thermal techniques can provide rapid and reliable information regarding lipid organization, oxidative stability, antioxidant content, and chemical composition.

Overall, the findings reinforce the direct relationship between degree of unsaturation and oxidative stability, demonstrating that oils rich in monounsaturated fatty acids exhibit greater thermo-oxidative resistance, whereas highly polyunsaturated or terpene-rich oils are more susceptible to oxidative degradation. These results contribute to a broader understanding of the physicochemical behavior of vegetable and essential oils and may support future applications related to quality control, authentication, industrial processing, and evaluation of oxidative stability in lipid-based systems.

## Figures and Tables

**Table 1:** DSC-derived thermal properties of vegetable oils, including peak temperatures and enthalpy values for cooling and heating cycles.

Oil Type	Curve	Peak 1			Peak 2		
		Initial Temperature (°C)	Peak Max Temperature (°C)	Enthalpy (W/g)	Initial Temperature (°C)	Peak Max Temperature (°C)	Enthalpy (W/g)
Avocado	Cool	−14.46	−20.36	4.372	−51.07	−58.42	13.29
	Heat	−30.56	−8.83	59.15	—	—	—
Eucalyptus A	Cool	−54.26	−53.65	41.91	—	—	—
	Heat	−28.3	−3.90	37.16	—	—	—
Eucalyptus B	Cool	−50.28	−50.83	51.65	—	—	—
	Heat	−25.87	−3.02	44.09	—	—	—
Orange A	Cool	—	—	—	—	—	—
	Heat	—	—	—	—	—	—
Orange B	Cool	—	—	—	—	—	—
	Heat	—	—	—	—	—	—
Sucupira A	Cool	−11.46	−19.89	9.375	—	—	—
	Heat	−39.67	−33.81	3.864	−27.99	−18.48	32.54
Sucupira B	Cool	−13.67	−19.44	6.762	—	—	—
	Heat	−38.16	−31.97	1.093	−21.80	−4.65	3.756
Grape seed A	Cool	−12.43	−18.81	9.915	—	—	—
	Heat	−39.14	−32.97	4.487	−27.11	−16.96	40.57
Grape seed B	Cool	−15.26	−24.60	2.355	—	—	—
	Heat	−51.87	−26.19	42.65	—	—	—

Note: — indicates a non-detectable result; values represent the principal thermal parameters extracted from representative DSC curves obtained from three repeated measurements of the same sample.

**Table 2:** Fatty acid profile of oils obtained in this study.

	Avocado	Eucalyptus A and B	Orange A and B	Sucupira A	Sucupira B	Grape Seed A	Grape Seed B
Saturated Fatty Acids (SFA)	Composition (%)						
butyric (C4:0)	—	13.23	1.5	—	—	—	—
Caproic (C6:0)	—	86.72	93.64	—	—	—	—
Caprylic (C8:0)	0.12	—	—	—	—	—	—
Capric (C10:0)	0.12	—	0.57	3.22	0.11	—	—
Undecanoic (C11:0)	—	0.02	0.3	—	—	—	—
Lauric (C12:0)	1.82	—	1.76	—	—	—	—
Tridecanoic (C13:0)	—	—	0.12	1.33	—	—	—
Myristic (C14:0)	0.68	—	0.51	0.16	0.09	0.12	—
Pentadecanoic (C15:0)	—	—	0.54	0.33	—	—	—
Palmitic (C16:0)	7.35	—	0.08	11.18	10.70	10.79	6.46
margaric (C17:0)	—	—	—	0.27	—	—	—
stearic (C18:0)	2.34	—	—	4.32	3.78	3.81	4.38
arachidic (C20:0)	0.01	—	—	0.02	0.02	0.04	0.33
Heneicosanoic (C21:0)	0.03	—	—	0.06	0.05	0.07	—
behenic (C22:0)	—	—	—	0.14	—	—	—
Tricosanoic (C23:0)	—	—	—	0.02	—	—	—
lignoceric (C24:0)	0.05	—	—	0.92	0.27	—	—
∑SFA	12.52	99.97	99.02	21.97	15.02	14.83	11.17
**Monounsaturated** **fatty**							
Myristoleic (C14:1)	—	—	0.73	—	—	—	—
Palmitoleic (C16:1)	1.87	—	—	0.31	0.19	0.2	—
Margaroleic (C17:1)	—	—	0.02	0.06	—	—	—
Elaidic (C18:1n9t)	—	0.03	0.04	24.27	23.66	26.55	13.33
Oleic (C18:1n9c)	60.63	—	0.02	1.24	1.47	1.51	0.54
Eicosenoic (C20:1)	0.54	—	—	0.29	0.29	0.28	0.24
Erucic (C22:1n9)	—	—	—	0.07	—	—	—
Nervoni (C24:1n9)	0.11						
∑MUFA	63.15	0.03	0.81	26.24	25.61	28.36	14.11
**Polyunsaturated fatty acids**							
Linoleic (C18:2n6c)	15.96	—	0.12	45.3	52.57	50.46	66.84
Linolelaidic (C18:2n6t)	0.04	—	—	—	—	—	0.93
γ—Linolenic (C18:3n6)	0.23	—	0.03	0.20	0.38	0.31	0.39
α—Linolenic (C18:3n3)	3.37	—	0.01	4.93	5.86	5.83	1.31
Eicosadienoic (C20:2)	0.02	—	—	0.03	0.03	0.03	0.03
Arachidonic (C20:4n6)	0.51	—	—	0.41	0.43	—	0.15
Eicosapentaenoic (C20:5n3)	0.03	—	—	0.29	0.06	—	—
Dihomo-γ-linolenic acid (C20:3n6)	—	—	0.03	—	—	—	—
Docosadienoic (C22:2)	—	—	—	0.08	0.04	—	0.2
∑PUFA	20.16	—	0.19	51.24	59.37	56.63	69.85

Note: — indicates a nondetectable result; fatty-acid composition corresponds to the analytical determination of each oil sample. Repeated injections of the same prepared sample were performed to verify chromatographic repeatability.

**Table 3:** Oxidative stability parameters of the evaluated oils, including induction temperature and induction time.

Oils	Temperature	Induction Period
Avocado	110°	12.31 h
Eucaliptus A e B	110°	0.28 h
Orange A e B	110°	0.02 h
Sucupira A	110°	0.02 h
Sucupira B	110°	0.01 h
Grape seed A	110°	6.18 h
Grape seed B	110°	0.04 h

## Data Availability

The data used to support the findings of this study are available from the corresponding author upon request.

## Abbreviations

The following abbreviations are used in this manuscript:

DSC	differential scanning calorimetry
GC-FID	gas chromatography
SFA	saturated fatty acids
MUFA	monounsaturated fatty acids
PUFA	polyunsaturated fatty acids
EEMs	Excitation–emission fluorescence matrices
OSI	oxidative stability index

## References

[1] Gunstone F.D. (2011). Vegetable Oils in Food Technology: Composition, Properties and Uses.

[2] Shahidi F., Zhong Y. (2010). Lipid oxidation and improving the oxidative stability. Chem. Soc. Rev..

[3] Bakkali F., Averbeck S., Averbeck D., Idaomar M. (2008). Biological effects of essential oils—A review. Food Chem. Toxicol..

[4] Burt S. (2004). Essential oils: Their antibacterial properties and potential applications in foods—A review. Int. J. Food Microbiol..

[5] Fernandes C.D.P., Pott A., Hiane P.A., Nascimento V.A., Filiú W.F.O., Oliveira L.C.S., Sanjinez-Argandoña E.J., Cavalheiro L.F., Nazário C.E.D., Caires A.R.L. (2024). Comparative analysis of grape seed oil, linseed oil, and a blend: In vivo effects of supplementation. Foods.

[6] Flores M., Saravia C., Vergara C.E., Avila F., Valdés H., Ortiz-Viedma J. (2019). Avocado oil: Characteristics, properties, and applications. Molecules.

[7] Wang W., Bostic T.R., Gu L. (2010). Antioxidant capacities, procyanidins and pigments in avocados of different strains and cultivars. Food Chem..

[8] Elaissi A., Rouis Z., Mabrouk S., Salem N.A.B., Aouni M., Farhat F., Chemli R., Harzallah-Skhiri F., Khouja M.L. (2012). Correlation between chemical composition and antibacterial activity of essential oils from fifteen *Eucalyptus* species growing in Tunisia. Molecules.

[9] Geraci A., Di Stefano V., Di Martino E., Schillaci D., Schicchi R. (2017). Essential oil components of orange peels and antimicrobial activity. Nat. Prod. Res..

[10] Razola-Díaz M.C., Guerra-Hernández E., García-Villanova B., Verardo V. (2021). Recent developments in extraction and encapsulation techniques of orange essential oil. Food Chem..

[11] Ferreira S.B., de Oliveira Q.F., de Souza A.M.T., de Carvalho M.G. (2014). Chemical constituents from *Pterodon emarginatus*. Rev. Bras. Farmacogn..

[12] Costa-Singh T., Jorge N. (2019). Characterization of seed oils of *Pterodon emarginatus* Vogel (sucupira) obtained by different extraction methods. Braz. Arch. Biol. Technol..

[13] Rockenbach I.I., Gonzaga L.V., Rizelio V.M., Gonçalves A.E.S.S., Genovese M.I., Fett R. (2011). Phenolic compounds and antioxidant activity of seed and skin extracts of red grape varieties. Food Res. Int..

[14] Garavaglia J., Markoski M.M., Oliveira A., Marcadenti A. (2016). Grape seed oil compounds: Biological and chemical actions for health. Nutr. Metab. Insights.

[15] Choe E., Min D.B. (2006). Mechanisms and factors for edible oil oxidation. Compr. Rev. Food Sci. Food Saf..

[16] Melo M.A., Michels F., Arakaki D., Lima N., Gonçalves D., Cavalheiro L., Oliveira L., Caires A., Hiane P., Nascimento V. (2019). First study on the oxidative stability and elemental analysis of babassu (*Attalea speciosa*) edible oil produced in Brazil using a domestic extraction machine. Molecules.

[17] Christie W.W. (1989). Gas Chromatography and Lipids: A Practical Guide.

[18] Tan C.P., Che Man Y.B. (2002). Differential scanning calorimetric analysis of edible oils. Food Chem..

[19] Metrohm Application Bulletin (2010). Rancimat Method for Oxidative Stability Determination.

[20] Sikorska E., Khmelinskii I., Sikorski M. (2012). Fluorescence spectroscopy in edible oil analysis. Food Chem..

[21] Guillén M.D., Cabo N. (1997). Characterization of edible oils and lard by Fourier transform infrared spectroscopy. Food Chem..

[22] Albuquerque M.L.S., Guedes I., Alcantara P., Moreira S.G.C., Barbosa Neto N.M., Correa D.S., Zilio S.C. (2005). Characterization of buriti (*Mauritia flexuosa* L.) oil by absorption and emission spectroscopies. J. Braz. Chem. Soc..

[23] Salgado J.M., Danieli F., Regitano-d’Arce M.A.B., Frias A., Mansi D.N. (2008). The avocado oil quality and stability. Food Sci. Technol..

[24] Bail S., Stuebiger G., Krist S., Unterweger H., Buchbauer G. (2008). Characterisation of various grape seed oils by volatile compounds, triacylglycerol composition, total phenols and antioxidant capacity. Food Chem..

[25] Torres M.P.R., Guia M.R.Z., Morales R.L., Valladão D.M.S. (2026). Characterization of the essential oil extracted from the fruits of sucupira. Nativa.

[26] Turek C., Stintzing F.C. (2013). Stability of essential oils: A review. Compr. Rev. Food Sci. Food Saf..

[27] Guerrero F., Carmona C., Hernández C., Toledo M., Arriagada A., Espinoza L., Bergmann J., Taborga L., Yañez K., Carrasco Y. (2022). Drivers of flammability of *Eucalyptus globulus* Labill. leaves: Terpenes, essential oils, and moisture content. Forests.

[28] Chaiyasut C., Kesika P., Sirilun S., Makhamrueang N., Peerajan S., Sivamaruthi B.S. (2019). Influence of extraction process on yield, total phenolic content, and antioxidant properties of avocado (*Persea americana* Mill.) oil and stability assessment. Asian J. Pharm. Clin. Res..

[29] Aktar T., Adal E. (2019). Determining the Arrhenius kinetics of avocado oil: Oxidative stability under Rancimat test conditions. Foods.

[30] Maszewska M., Florowska A., Dłużewska E., Wroniak M. (2018). Oxidative stability of selected edible oils. Molecules.

[31] Passos W.E., Segovia M.V.A., Lescano C.H., Oliveira I.P., Caires A.R.L., Muzzi R.M. (2025). Absorption and fluorescence profiles of biodiesel blends from kernel and pulp oils: Optical responses of α-tocopherol and β-carotene under thermal degradation. J. Am. Oil Chem. Soc..

[32] Sikorska E., Górecki T., Khmelinskii I.V., Sikorski M., Koziol J. (2008). Fluorescence spectroscopy in monitoring of edible oils. Optical Monitoring of Fresh and Processed Agricultural Crops.

[33] Anwar F., Hussain A.I., Iqbal S., Bhanger M.I. (2007). Enhancement of the oxidative stability of some vegetable oils by blending with *Moringa oleifera* oil. Food Chem..

[34] Wang M., Yu P., Chittiboyina A.G., Chen D., Zhao J., Avula B., Wang Y.H., Khan I.A. (2020). Characterization, quantification and quality assessment of avocado (*Persea americana* Mill.) oils. Molecules.

[35] Bligh E.G., Dyer W.J. (1959). A rapid method of total lipid extraction and purification. Can. J. Biochem. Physiol..

[36] Hartman L., Lago R.C.A. (1973). Rapid preparation of fatty acid methyl esters from lipids. Lab. Pract..

[37] Maia E.L., Rodriguez-Amaya D.B. (1993). Avaliação de um método simples e rápido para metilação de ácidos graxos com lipídios de alimento. Rev. Inst. Adolfo Lutz.

[38] (2020). Fat and Oil Derivatives—Fatty Acid Methyl Esters (FAME)—Determination of Oxidation Stability (Accelerated Oxidation Test).

